# Intracellular Alkalinization Induces Cytosolic Ca^2+^ Increases by Inhibiting Sarco/Endoplasmic Reticulum Ca^2+^-ATPase (SERCA)

**DOI:** 10.1371/journal.pone.0031905

**Published:** 2012-02-27

**Authors:** Sen Li, Baixia Hao, Yingying Lu, Peilin Yu, Hon-Cheung Lee, Jianbo Yue

**Affiliations:** Department of Physiology, University of Hong Kong, Hong Kong, China; University of Georgia, United States of America

## Abstract

Intracellular pH (pHi) and Ca^2+^ regulate essentially all aspects of cellular activities. Their inter-relationship has not been mechanistically explored. In this study, we used bases and acetic acid to manipulate the pHi. We found that transient pHi rise induced by both organic and inorganic bases, but not acidification induced by acid, produced elevation of cytosolic Ca^2+^. The sources of the Ca^2+^ increase are from the endoplasmic reticulum (ER) Ca^2+^ pools as well as from Ca^2+^ influx. The store-mobilization component of the Ca^2+^ increase induced by the pHi rise was not sensitive to antagonists for either IP_3_-receptors or ryanodine receptors, but was due to inhibition of the sarco/endoplasmic reticulum Ca^2+^-ATPase (SERCA), leading to depletion of the ER Ca^2+^ store. We further showed that the physiological consequence of depletion of the ER Ca^2+^ store by pHi rise is the activation of store-operated channels (SOCs) of Orai1 and Stim1, leading to increased Ca^2+^ influx. Taken together, our results indicate that intracellular alkalinization inhibits SERCA activity, similar to thapsigargin, thereby resulting in Ca^2+^ leak from ER pools followed by Ca^2+^ influx via SOCs.

## Introduction

The activity of virtually all proteins and macromolecules can be modulated by protons; thus intracellular pH (pHi) is rigorously regulated for survival [1], [2], [3]. Subtle and transient pHi changes occur under many physiological conditions. For examples, activity-dependent membrane depolarization elevates pHi in astrocytes of rat cortex [4]. Likewise, both capacitation of spermatozoa [5] and fertilization of eggs [6], induce intracellular alkalinization. Much greater and sustained pHi changes, on the other hand, can occur under pathological conditions, e.g. acidification of pHi during apoptosis and alkalinization in tumorigenesis [2]. Cells passively stabilize pHi by the buffering capacity of a variety of intracellular weak acids and bases, especially HCO_3_^−^, generated by CO_2_ hydration and subsequent deprotonation of carbonic acid. However, these intrinsic buffering systems can be overpowered during continued extra- and intracellular stress or stimulation. Cells, therefore, have evolved a complicated proton transporting system to regulate cytosolic pH as well as the pH in other cellular compartments [1].

Under physiological conditions, cells utilize two major pH-regulatory ion transporters at the plasma membrane, the Na^+^-H^+^-exchangers (NHEs) and the Na^+^-HCO_3_^−^ co-transporters (NBCs), to extrude the protons produced during normal cellular metabolic activity. Some cells also utilize Na^+^-dependent Cl^−^-HCO_3_^−^ exchangers (NDCBEs) or monocarboxylate-H^+^ co-transporters (MCTs) for similar purposes. On the other hand, activation of the Cl^−^-HCO_3_^−^ anion exchanger (AEs) and plasma membrane Ca^2+^-ATPase (PMCAs) can lead to cytosolic acidification. Among all of these transporters, NHE1 is perhaps the most dominant one for maintaining pHi homeostasis [1], [2]. Its activity is not only modulated by cytosolic H^+^, but also by various extra- or intra-cellular signals, leading to elevation of pHi during diverse cellular processes, such as autophagy, migration, adhesion, chemotaxis, and cell cycle progression [2], [7], [8].

Likewise, Ca^2+^ is equally important in regulating diverse cell functions, including fertilization, cell proliferation and differentiation [9]. Cytoplasmic Ca^2+^ level at rest is kept low mainly by the active extrusion of cytosolic Ca^2+^ out of cells via the PMCAs and the Na^+^/Ca^2+^ -exchanger, as well as by sequestration of Ca^2+^ into the endoplasmic reticulum (ER) and mitochondria via a sarco/endoplasmic reticulum Ca^2+^-ATPase (SERCA) and a mitochondria Ca^2+^ uniporter, respectively. External signaling can markedly increase cytoplasmic Ca^2+^ levels by opening plasma membrane ion channels, such as voltage-gated Ca^2+^-selective channels (CaVs) and transient receptor potential (TRP) ion channels. Cytoplasmic Ca^2+^ concentration can also be suddenly and dramatically increased by release from the ER Ca^2+^ store, through activation of IP_3_ receptors (IP_3_Rs) and ryanodine receptors (RyRs) in ER [10]. At least three endogenous Ca^2+^ mobilizing messengers have been identified for regulating these Ca^2+^ release channels, which include inositol trisphosphate (IP_3_), nicotinic acid adenine dinucleotide phosphate (NAADP), and cyclic adenosine diphosphoribose (cADPR). Ca^2+^ influx and internal Ca^2+^ store release are normally interconnected. Thus, depletion of the ER Ca^2+^ store can trigger activation of the store-operated channels (SOCs) at the plasma membrane, mediated by Orails and Stims, and lead to Ca^2+^ influx [11].

Intracellular alkalinization has been linked to intracellular Ca^2+^ events. Intracellular Ca^2+^ spikes that occur during oocyte maturation and egg fertilization in several marine invertebrate and amphibian species are believed to be responsible for regulating the subsequent alkalinization of pHi [12]. Conversely, intracellular alkalinization occurring during the initiation of sperm motility and the activation of the acrosome reaction can likewise regulate Ca^2+^ uptake [5], [13]. In vertebrates, intracellular alkalinization can indeed increase Ca^2+^ current in a wide variety of cells and tissues, including various types of neurons[14], [15], rat pancreatic acinar cells [16], different types of muscle cells [17], mast cells [18], aortic endothelial cells [19], and lymphocytes [20]. Different membrane Ca^2+^ channels are involved in alkaline pHi-triggered Ca^2+^ entry depending on cell or tissue types [5], [15], [21], [22], which have been well documented. However, the studies on the mechanism of alkaline pHi triggered internal Ca^2+^ release are sparse and conflicting, especially concerning whether or how IP_3_ signaling is required [19], [23], [24].

Here we studied the mechanisms underlying intracellular alkalinization-induced cytosolic Ca^2+^ changes in various cell lines. We demonstrated that intracellular alkalinization inhibits SERCA activity, leading to Ca^2+^ leak from the ER. The depletion of ER Ca^2+^ stores then activates Ca^2+^ influx through SOCs of Stim1 and Orai1.

## Results

### Intracellular alkalinization induces cytosolic Ca^2+^ increase

**Figure 1:**
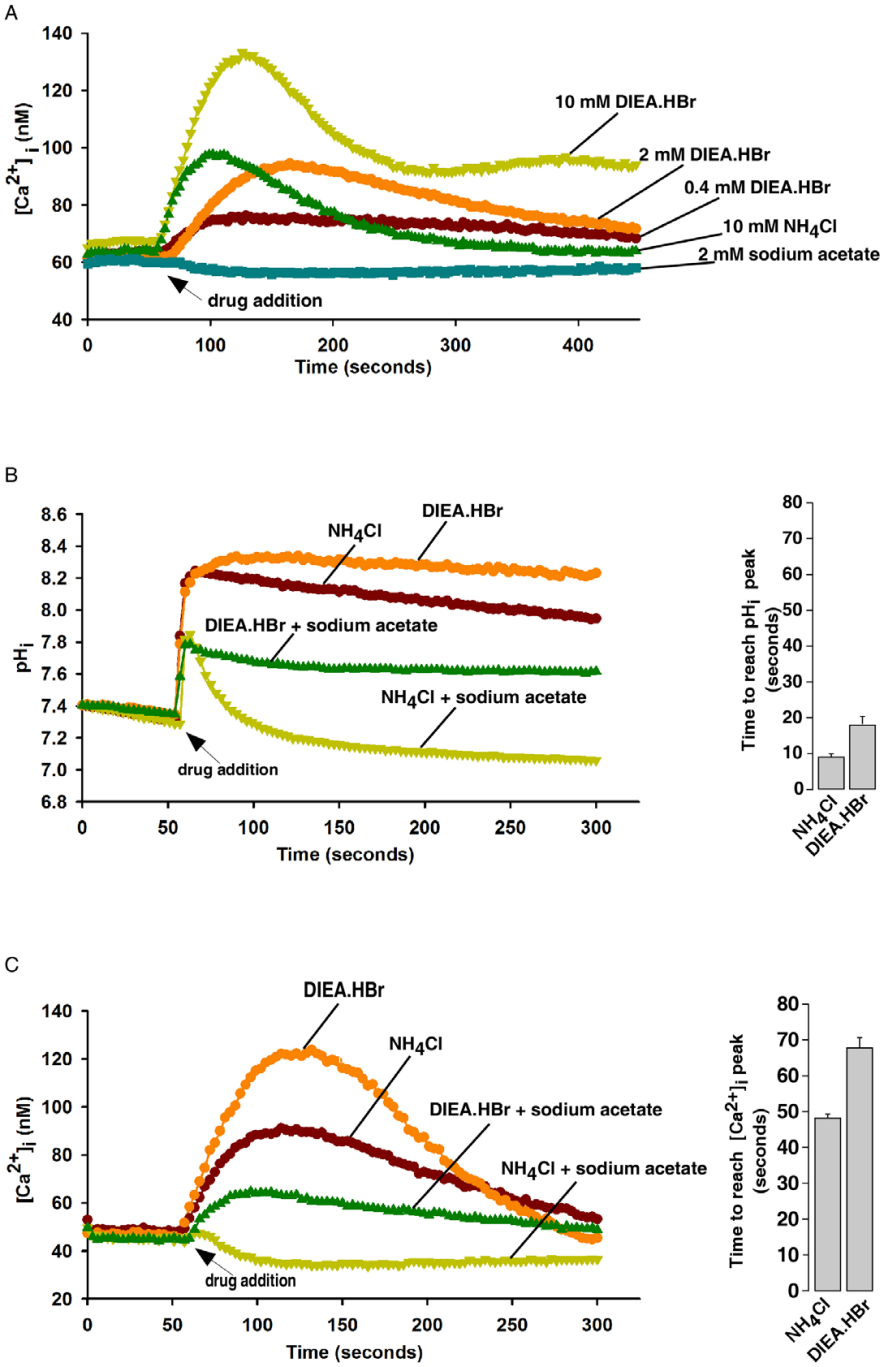
Intracellular alkalinization induces cytosolic Ca^2+^ increases in HeLa cells. (A) DIEA.HBr, similar to NH_4_Cl, induced cytosolic Ca^2+^ increases in a dose-dependent manner in HeLa cells as measured by the Ca^2+^-indicator, Fura-2 AM. (B) Intracellular alkalinization induced by DIEA.HBr (10 mM) and NH_4_Cl (10 mM) were inhibited by sodium acetate (40 mM) as measured by the pH-indicator, BCECF AM. (C) Cytosolic Ca^2+^ increases induced by DIEA.HBr (10 mM) and NH_4_Cl (10 mM) were markedly inhibited by sodium acetate (40 mM). The graphs represent data from three independent experiments. Data quantifications of the time to reach pH_i_ peak (B) or [Ca^2+^]_i_ peak (C) after drug treatment were expressed as mean ± S.E., n = 30–50 cells.

It has been shown previously that weak bases, such as ammonium chloride (NH_4_Cl) or methylamine, can induce cytosolic Ca^2+^ increase (reviewed in Ref. [3]). However, the high concentrations of these bases used in these studies complicated the interpretation of the results due to osmolarity changes or impurity of the compounds. In the process of synthesizing cell permeant analogs of the Ca^2+^ releasing messangers IP_3_, NAADP, and cADPR, we found the hydrobromide salt of diisopropylethyl amine (DIEA.HBr), an organic base commonly used in the organic chemistry, can induce cytosolic Ca^2+^ increases, similar to that of NH_4_Cl, in a dose dependent manner, whereas sodium acetate, a weak acid, failed to change Ca^2+^ (Figure 1A). We purified DIEA.HBr by HPLC and crystallization (Figure S1). Controls showed that neither NaBr nor KBr could induce any cytosolic Ca^2+^ changes (Figure S2). In addition, DIEA.HBr induced cytosolic pH increase in a dose dependent manner in HeLa cells (Figure S3). As shown in Figures 1B and 1C, the pH rise triggered by DIEA.HBr or NH_4_Cl preceded the cytosolic Ca^2+^ increase. Moreover, administration of weak acids, such as sodium acetate, markedly inhibited the ability of DIEA.HBr or NH_4_Cl to induce intracellular alkalinization and cytosolic Ca^2+^ rises. Similar results have been observed in various cell lines, showing generality (Figure S4). These results thus indicate that intracellular alkalinization induces cytosolic Ca^2+^ increase.

### Intracellular alkalinization induces Ca^2+^ release from ER Ca^2+^ pools independent of IP_3_ receptors and ryanodine receptors

**Figure 2:**
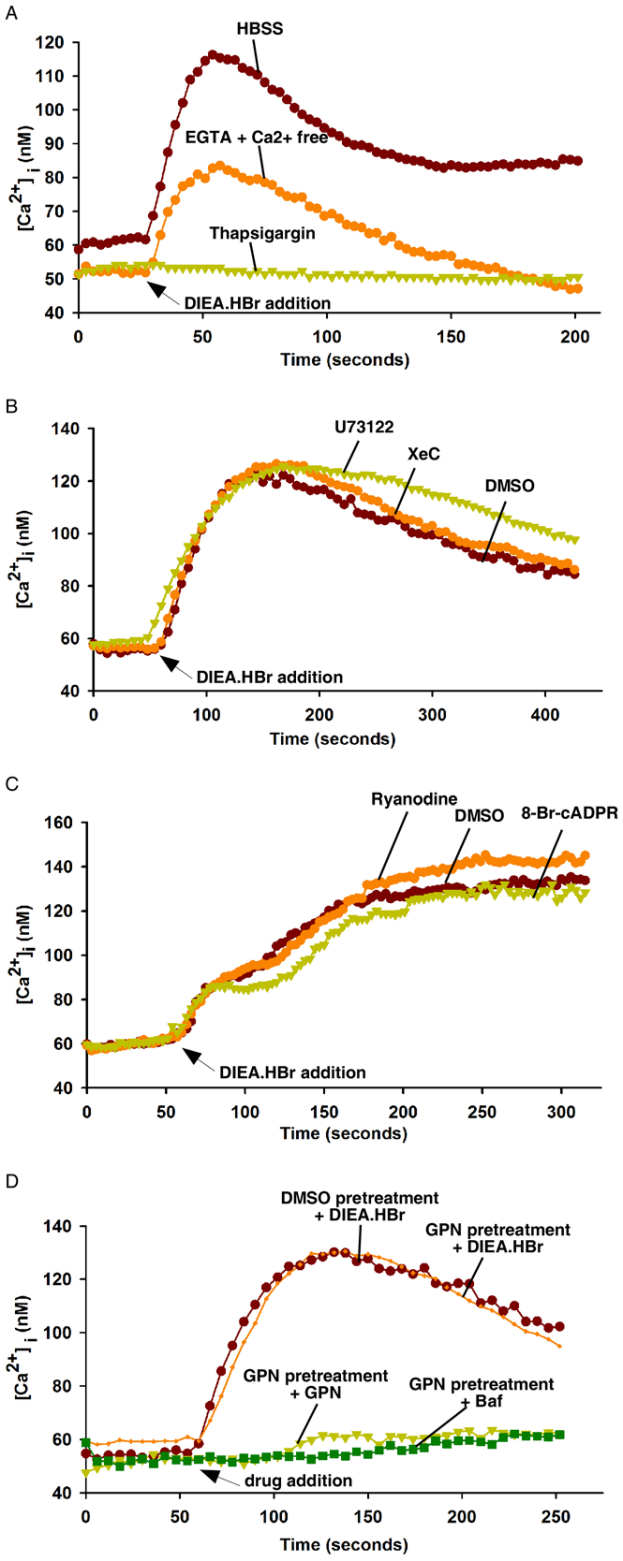
Intracellular alkalinization releases Ca^2+^ from ER pools in HeLa cells and PC12 cells. (A) DIEA.HBr (4 mM)-induced Ca^2+^ increase in Fura-2 loaded HeLa cells was abolished by thapsigargin (1 µM) pretreatment. This Ca^2+^ increase was inhibited by removal of external Ca^2+^ (Ca^2+^-free HBSS with 4 mM EGTA). (B) Pretreatment of Fura-2 loaded HeLa cells with either *Xestospongin C (XeC)* (10 µM) or U73122 (10 µM) did not inhibit the DIEA.HBr-induced Ca^2+^ increase compared with untreated cells. The graphs represent data from three independent experiments. (C) Pretreatment of Fura-2 loaded PC12 cells with ryanodine (20 µM) or 8-Br-cADPR (100 µM) did not inhibit the DIEA.HBr-induced Ca^2+^ increase compared with untreated cells. The graphs represent data from three independent experiments. (D) Pretreatment of Fura-2 loaded HeLa cells with glycyl-l-phenylalanine 2-naphthylamide (GPN) (50 µM) did not inhibit the DIEA.HBr-induced Ca^2+^ increase compared with untreated cells while completely blocked GPN or bafilomycin A1 (0.5 µM)-induced Ca^2+^ increase. The graphs represent data from three independent experiments.

Next, we traced the sources of the cytosolic Ca^2+^ increases induced by these bases. Since treatment of a variety of cell types with NH_4_Cl or DIEA.HBr basically generated similar results, only the data of DIEA.HBr in HeLa cells, PC12 cells, and NIH 3T3 cells were presented in the remainder of the results section. Pretreatment with thapsigargin, a specific SERCA inhibitor, completely abolished DIEA.HBr-induced Ca^2+^ changes in HeLa cells, and the inclusion of EGTA in a Ca^2+^ free medium markedly diminished the Ca^2+^ peaks of the sustained phase (Figure 2A). Similar results have been observed in NIH3T3 cells and PC12 cells (Figure S4). These results indicate that intracellular alkalinization induces Ca^2+^ release from ER pools, which is followed by extracellular Ca^2+^ influx. Interestingly, treatment of HeLa cells with *Xestospongin C* (*XeC*), an IP_3_R antagonist, or U73122, a specific inhibitor of phospholipase C, had little effect on cytosolic Ca^2+^ increases induced by DIEA.HBr (Figure 2B). In contrast, *XeC* or U73122 effectively inhibited histamine-induced Ca^2+^ increases in HeLa cells (Figure S5A). Similar data have also been observed in several other cell lines (data not shown). These data indicate that the Ca^2+^ release from ER induced by intracellular alkalinization is independent of IP_3_Rs. In addition, no RyRs were detected in HeLa cells and HeLa cells were not responsive to caffeine treatment (data not shown and Figure S5B), suggesting that intracellular alkalinization-induced Ca^2+^ increases in HeLa cells is also independent of RyRs. Indeed, a high concentration of ryanodine, acting as a RyRs antagonist, or 8-Br-cADPR, a cADPR antagonist, had little effect on cytosolic Ca^2+^ increases induced by DIEA.HBr in PC12 cells (Figure 2C), whereas this concentration of ryanodine effectively inhibited caffeine-induced Ca^2+^ increases in PC12 cells (Figure S5B). We have also previously shown that 8-Br-cADPR can effectively block Ca^2+^ increases induced by cADPR, which reportedly mobilizes Ca^2+^ via RyRs from ER, in PC12 cells [25]. Similar results have been observed in several other RyRs-expressing cell lines (data not shown). These results document that the Ca^2+^ release from ER induced by intracellular alkalinization is independent of RyRs as well.

### Intracellular alkalinization induces cytosolic Ca^2+^ increase independent of acid Ca^2+^ store

To test whether the acidic Ca^2+^ store is affected by intracellular alkalinization, we treated HeLa cells with glycyl-l-phenylalanine 2-naphthylamide (GPN) to selectively disrupt the lysosomal membrane [26], and released the lysosomal Ca^2+^ [27]. As shown in Figure S6, 50 µM GPN completely depleted the lysosomal Ca^2+^ pools, evidenced by the fact that subsequent addition of GPN (50 µM) or bafilomycin A1 (0.5 µM), a specific inhibitor of the vacuolar-type H(+)-ATPase that is known to be able to release Ca^2+^ from the lysosomes normally, failed to release any more Ca^2+^. In contrast, pretreating cells with GPN failed to significantly alter the DIEA.HBr-induced Ca^2+^ rise in HeLa cells (Fiure 2D), indicating that the Ca^2+^ pools targeted by DIEA.HBr are not the lysosomes.

### Intracellular alkalinization releases Ca^2+^ from ER by lowering SERCA activity

**Figure 3:**
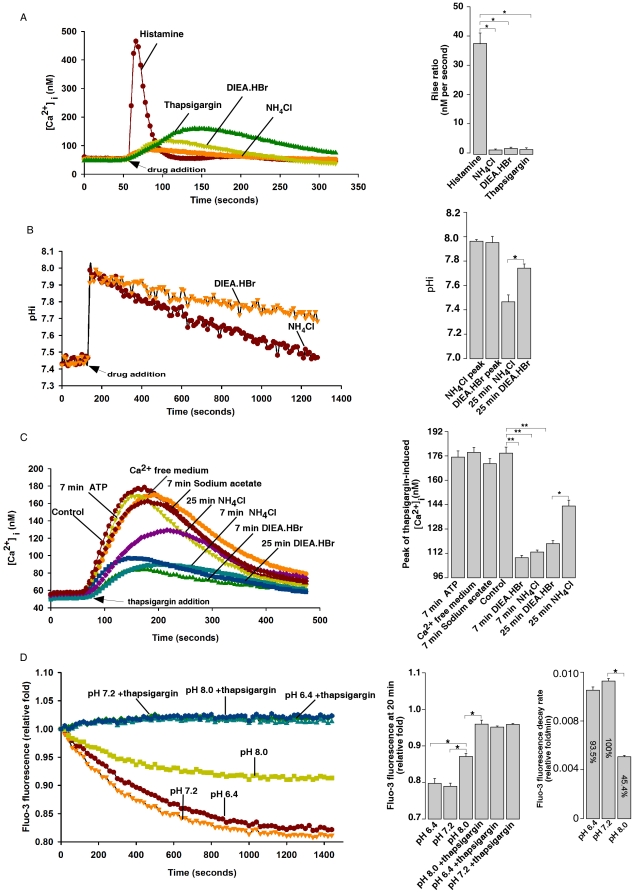
Intracellular alkalinization inhibits ER SERCA activity in HeLa cells. (A) Kinetics of cytosolic Ca^2+^ increases induced by histamine (10 µM), NH_4_Cl (4 mM), DIEA.HBr (4 mM), and thapsigargin (1 µM) in Fura-2 loaded HeLa cells. Data quantifications of rise ratio (340/380 per seconds) after drug treatment were expressed as mean ± S.E., n = 30–50 cells. The * symbols indicate the results of *t* Test analysis, *p*<0.05, compared with cells treated with histamine. (B) Kinetics of pHi changes induced by DIEA.HBr (4 mM) and NH_4_Cl (4 mM) in HeLa cells. Data quantifications of indicated pHi changes after drug treatment were expressed as mean ± S.D., *p*<0.05. (C) ER Ca^2+^ concentration, indicated by the thapsigargin (10 µM)-induced Ca^2+^ increase, was inhibited by pretreatment of Fura-2 loaded HeLa cells with DIEA.HBr (4 mM) or NH_4_Cl (4 mM) for 7 min or 25 min, but was not affected by ATP (100 µM) or sodium acetate (4 mM) pretreatment. Quantifications of thapsigargin-induced Ca^2+^ peaks were expressed as mean ± S.E., n = 30–50 cells, *p*<0.05 (*) or *p*<0.01 (**). (D) Alkaline pH inhibited Ca^2+^ uptake capability, whereas thapsigargin (1 µM) abolished Ca^2+^ uptake in Fluo-3 loaded permeabilized HeLa cells. Quantifications of Fluo-3 fluorescence at 20 min after drug additions and the decay rate of Fluo-3 fluorescence were expressed as mean ± S.D., *p*<0.05. All graphs represent data from three independent experiments.

The kinetics of Ca^2+^ release from ER by intracellular alkalinization markedly differed from that induced by histamine, which is known to be mediated by IP_3_Rs, whereas it is similar to the thapsigargin- triggered Ca^2+^ release (Figure 3A). Thapsigargin blocks SERCA and thereby allows Ca^2+^ leak from the ER into the cytosol. Since SERCA activity is known to be pH-dependent in vitro [28], [29] and SERCA ATPase activities in alkaline buffers were significantly lower than that in neural pH buffer (Figure S7), we speculated that intracellular alkalinization might release Ca^2+^ from ER by inhibiting SERCA activity as well. We, therefore, examined the ER Ca^2+^ content in HeLa cells at varied time points after pretreatment with DIEA.HBr or NH_4_Cl. The peak value of thapsigargin-induced cytosolic Ca^2+^ currents is commonly used as an index for ER Ca^2+^ content [30]. As shown in Figure 3B, NH_4_Cl or DIEA.HBr abruptly increased pH_i_ to similar levels, which were followed by a slower return to basal value. The kinetics of the pHi-return in the DIEA.HBr-treated cells was much slower than that in NH_4_Cl treated cells. Accordingly, pretreatment of HeLa cells with either base markedly reduced the amplitudes of thapsigargin induced Ca^2+^ rise, with the inhibitory extents correlating with the pHi value induced by either base in a time dependent manner (Figure 3C). On the other hand, pretreatment of cells with a weak acid, sodium acetate, or incubating cells in a Ca^2+^ free medium, or pretreatment of cells with ATP, had little effect on thapsigargin-induced Ca^2+^ release from ER. Similarly, intracellular alkalization also inhibited the amplitude of ionomycin-induced Ca^2+^ release in a Ca^2+^ free medium (Figure S8). These results indicate that intracellular alkalinization reduces the ER Ca^2+^ contents.

To demonstrate that the reduction of ER Ca^2+^ content by intracellular alkalinization was through inhibition of the SERCA, Ca^2+^ uptake experiments were performed. Saponin permeabilized HeLa cells were incubated in an uptake medium buffered at different pH values, and the Ca^2+^ concentration in the medium was measured by Fluo-3. The decay of Fluo-3 fluorescent signal over time is an indicator of Ca^2+^ uptake back into the ER and thus reflects the SERCA activity (Figure 3D) [31]. The addition of thapsigargin in the neutral uptake medium completely abolished Ca^2+^ uptake, indicating that SERCA was solely responsible for the Ca^2+^ uptake. Consistently, the decay of Fluo-3 intensity at alkaline pH (pH 8.0) was markedly inhibited as compared to that in neutral (pH 7.2) or acidic (pH 6.4) media (Figure 3D). Addition of thapsigargin to cells in an alkaline pH medium also completely abolished the remaining Ca^2+^ uptake. Similar results have also been observed in uptake buffer containing ruthenium red to exclude the possibility of Ca^2+^ uptake into mitochondria (Figure S9). All these data indicated that SERCA activity was inhibited, at least partially, in alkaline medium.

**Figure 4:**
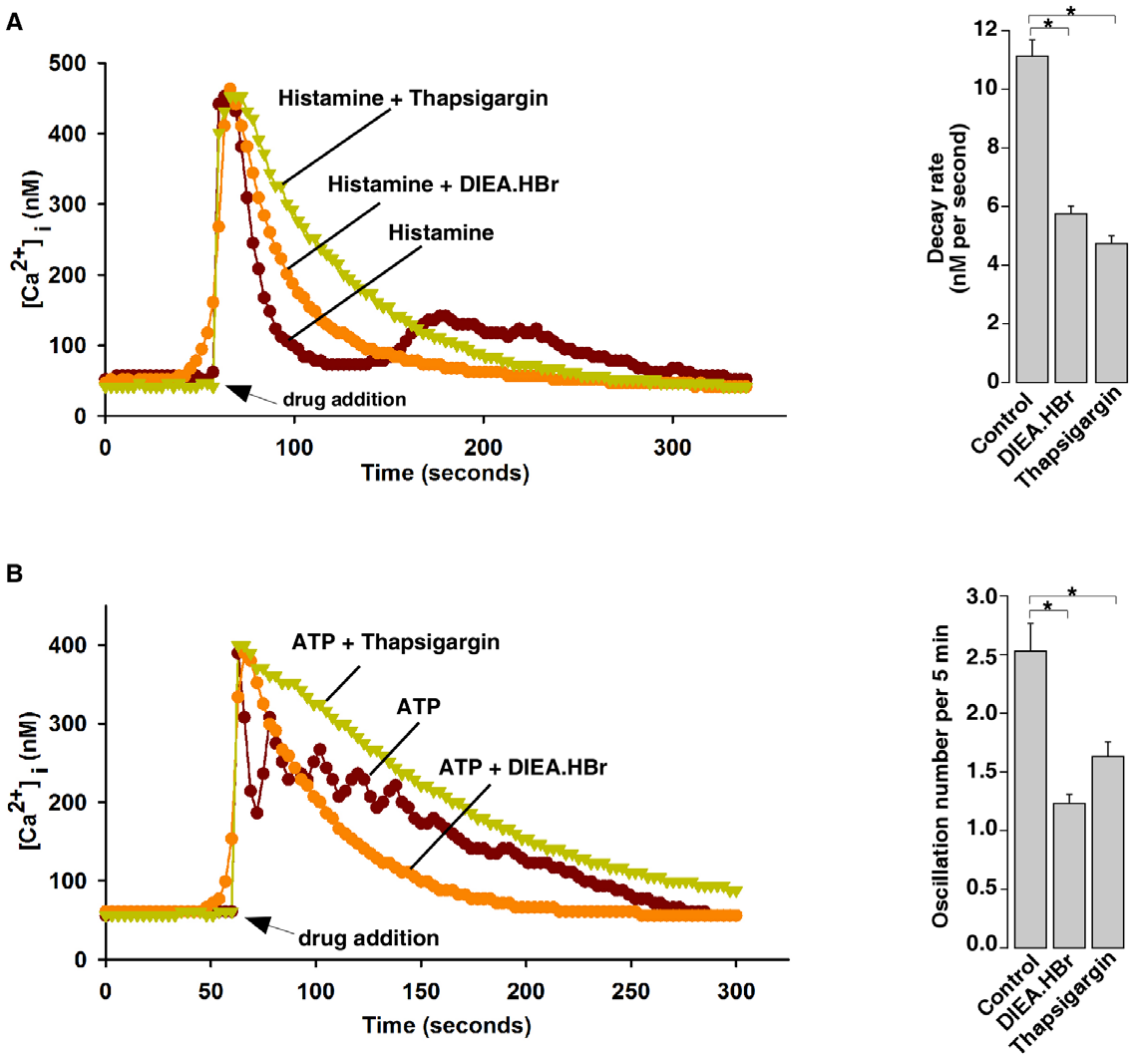
Intracellular alkalinization inhibits ER Ca^2+^ store filling after histamine and ATP treatment in HeLa cells. (A) Thapsigargin (10 µM) or DIEA.HBr (4 mM) inhibited ER Ca^2+^ refilling after histamine (10 µM) treatment in Fura-2 loaded HeLa cells. (B) Thapsigargin (10 µM) or DIEA.HBr (4 mM) diminished ATP (100 µM)-induced Ca^2+^ oscillations in Fura-2 loaded HeLa cells. The graphs represent data from three independent experiments, and data quantification are presented as mean ± S.E., n = 9–36 cells. The * symbols indicate the results of *t* Test analysis, *p*<0.05.

To further demonstrate that intracellular alkalinization inhibits SERCA activity, we examined the effects of intracellular alkalinization on sequestration of cytosolic Ca^2+^ to ER following Ca^2+^ release triggered by histamine or ATP in HeLa cells. In the absence of extracellular Ca^2+^, histamine releases Ca^2+^ from the ER into the cytosol via the IP_3_Rs, and SERCA then pumps the cytosolic Ca^2+^ back to the ER and returns the cytosolic Ca^2+^ to the basal levels. Thapsigargin abolishes SERCA activity, thereby greatly decreasing the decay rate of the cytosolic Ca^2+^. As showed in Figure 4A, the decay rate after histamine induced Ca^2+^ release was 11.1±0.9 nM/second (n = 36 cells), while this rate decreased to 4.9±0.3 nM/second (n = 29) in the presence of 10 µM thapsigargin. Similarly, the decay rate of histamine in the presence of DIEA.HBr was 5.8±0.4 nM/second (n = 9), which was also significant lower than that of histamine alone. Moreover, the effects of intracellular alkalinization on the decay rate of cytosolic Ca^2+^ after histamine treatment were examined in the presence of sodium orthovandate, a PMCA inhibitor (Figure S10B) [32], and similar results were observed (Figure S10A). Thus, the slower decay rate of cytosolic Ca^2+^ in alkaline pHi is due to the decreased sequestration of cytosolic Ca^2+^ to ER, not Ca^2+^ extrusion. In addition, it has previously been shown that the ER Ca^2+^ refilling via SERCA contributes to Ca^2+^ oscillations triggered by ATP in HeLa cells, since co-treatment with thapsigargin abolished the ATP-induced Ca^2+^ oscillation. We also found that intracellular alkalinization, similar to thapsigargin, blocked the ATP-induced Ca^2+^ oscillation (Figure 4B). Taken together, these data again demonstrated that intracellular alkalinization inhibits ER SERCA. It is noteworthy that histamine or ATP did not release more Ca^2+^, as indicated by the peak values of the fura 2 fluorescence, whether in the presence or absence of thapsigargin or DIEA.HBr (Figures 4A and 4B). Interestingly, adding thapsigargin (10 µM right after the peak of Ca^2+^ release triggered by DIEA.HBr generated another peak without reducing the subsequent Ca^2+^ decay rate, whereas adding DIEA.HBr after the peak of Ca^2+^ release evoked by thapsigargin (10 µM did not produce further Ca^2+^ release (Figure S11). Again, these data are consistent with the fact that alkalization partially inhibits SERCA, thereby leading to less extent of Ca^2+^ leak from ER than that by higher doses of thapsigargin (Figure 3A).

### Intracellular alkalinization induces Ca^2+^ influx by SOC pathway

**Figure 5:**
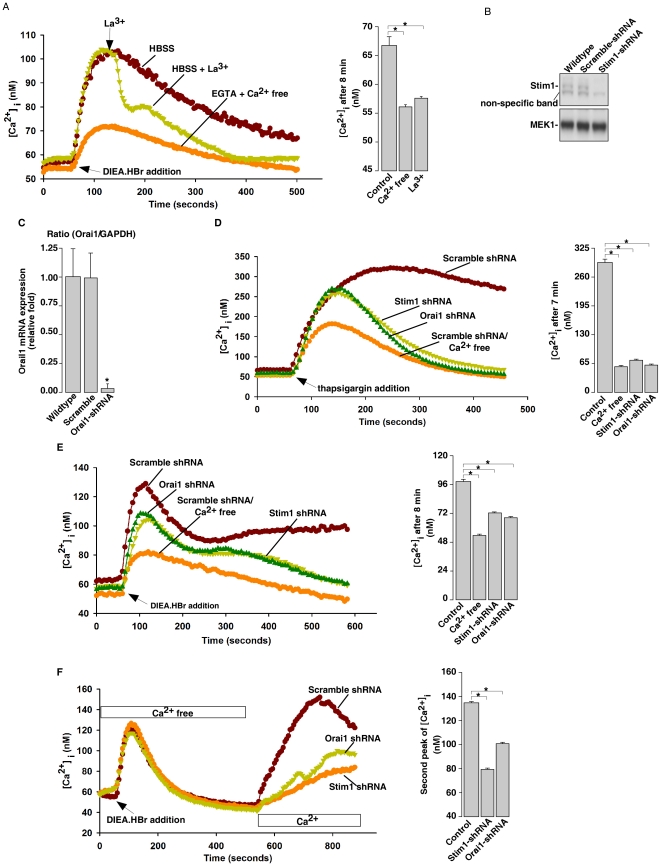
Intracellular alkalinization induces extracellular Ca^2+^ influx through SOCs in NIH 3T3 cells. (A) DIEA.HBr (4 mM) induced-Ca^2+^ influx was inhibited by La^3+^ (100 µM), a SOC blocker, treatment in Fura-2 loaded NIH3T3 cells incubated in regular HBSS. (B) Immunoblot analysis of Stim1-knockdown in NIH3T3 cells. MEK1 immunoblot was used as the internal control. (C) Quantitative real-time RT-PCR analysis of Orai1-knockdown in NIH3T3 cells. GAPDH was used as the internal control. Data are expressed as means ± S.D., n = 3. (D) and (E) Stim1 or Orai1 knockdown abolished the sustained Ca^2+^ influx induced by thapsigargin (10 µM) (D) and by DIEA.HBr (4 mM) (E) in Fura-2 loaded NIH3T3 cells incubated in regular HBSS. (F) Stim1 and Orai1 knockdown diminished the Ca^2+^ influx induced by DIEA.HBr (4 mM) in Fura-2 loaded NIH3T3 cells. Cells were initially treated with thapsigargin (1 µM) in Ca^2+^-free HBSS to deplete ER Ca^2+^ pool, followed by 2 mM Ca^2+^ addition. All graphs represent data from three independent experiments. Data quantification in (A), (D), (E), and (F) are presented as mean ± S.E., n = 30–50 cells. The * symbols indicate the results of *t* Test analysis, *p*<0.05.

**Figure 6:**
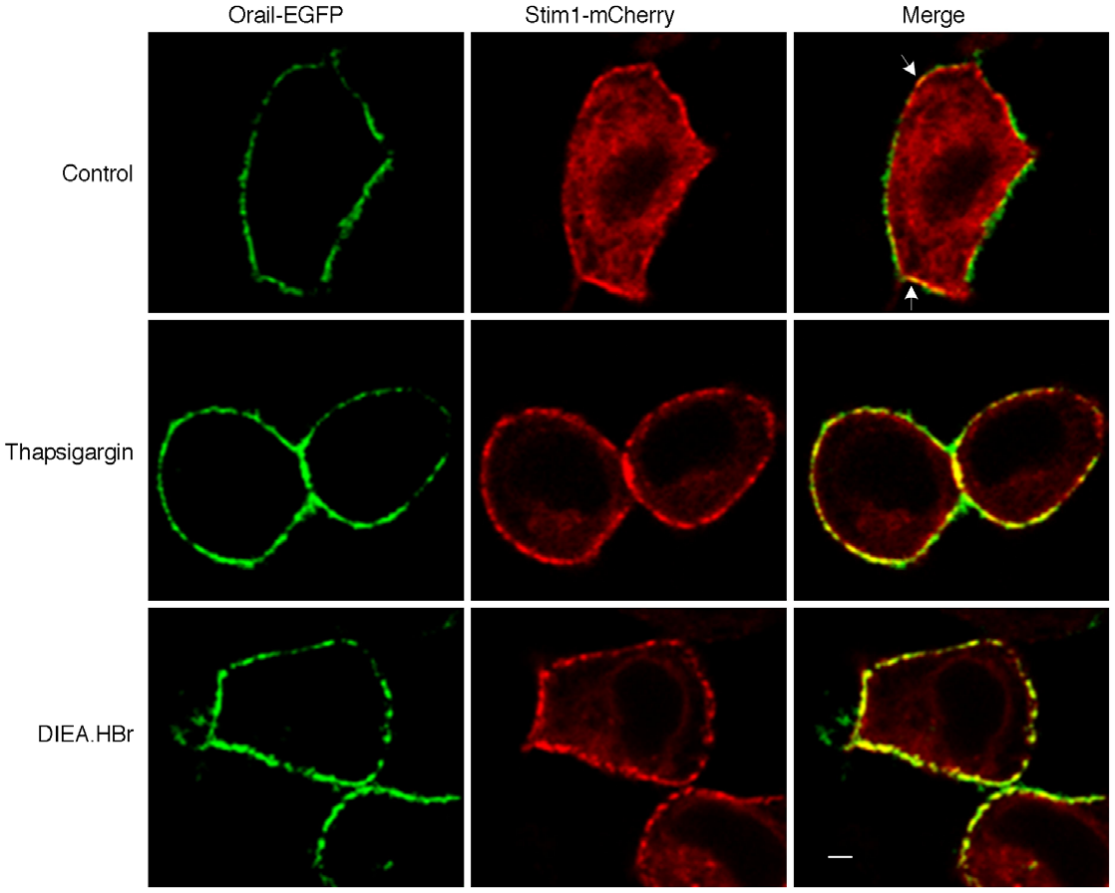
Intracellular alkalinization induces Stim1 and Orai1 colocalization in HeLa cells. HeLa cells, co-transfected with plasmids, Stim1-mCherry and Orai1-EGFP, were incubated in Ca^2+^ free HBSS for 15 min as control or treated with thapsigargin (10 M) or DIEA.HBr (4 mM) for 15 min in Ca^2+^ free HBSS. Confocal imaging of both mCherry and EGFP were taken. The graphs represent data from three independent experiments. Scale bar: 5 µm.

ER Ca^2+^ pool depletion could activate Stim-Orai-mediated SOCs to trigger Ca^2+^ influx [11]. Here we have shown above that Ca^2+^ influx contributes to the sustained Ca^2+^ phase of alkaline pHi-induced Ca^2+^ changes (Figure 2A). We, therefore, examined whether the Ca^2+^ influx triggered by intracellular alkalinization is via SOCs. Indeed, in NIH 3T3 cells, application of 100 µM La^3+^, an inhibitor of SOCs [33], at the peak of the DIEA.HBr-induced Ca^2+^ release in regular HBSS quickly returned Ca^2+^ to resting levels (Figure 5A). Similar results have also been observed in several other cell lines (data not shown). Since we have shRNAs against mouse Stim1 and Oria1 on hand, we next knocked down Stim1 or Orai1 in mouse NIH3T3 cells to further examine the role of SOCs in the intracellular alkalinization-induced Ca^2+^ influx (Figures 5B and 5C). As expected, thapsigargin (Figure 5D) or intracellular alkalization (Figures 5E and 5F)-induced Ca^2+^ influx was markedly inhibited in Stim1 or Orai1 knockdown cells as compared to that in the control cells. Moreover, when Orai1-EGFP and Stim1-mCherry were co-transfected into HeLa cells, confocal microscopy live cell imaging analysis showed that intracellular alkalinization, similar to thapsigargin, greatly induced the co-localization of Stim1 and Orai1 at the plasma membrane (Figure 6). Taken together, these data clearly indicate that intracellular alkalinization induces Ca^2+^ influx via SOCs of Orai1 and Stim1.

### Extracellular alkalinization induces a cytosolic Ca^2+^ increase

**Figure 7:**
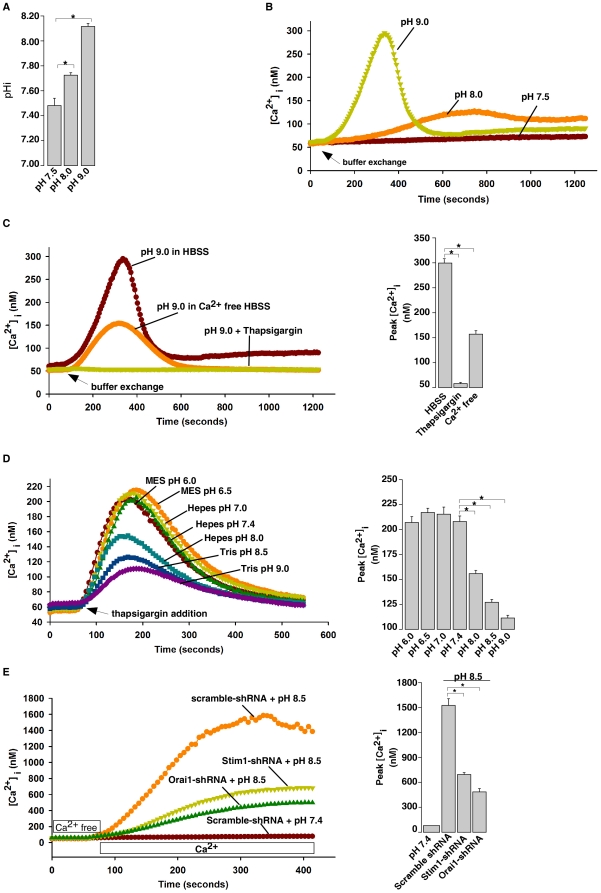
Extracellular alkalinization induces cytosolic Ca^2+^ increases in μHeLa cells and NIH3T3 cells. (A) Extracellular alkaline buffers induced pHi rise in HeLa cells as measured by the pH-indicator, BCECF AM. Data are expressed as means ± S.D., n = 3. (B) Extracellular alkaline buffers induced cytosolic Ca^2+^ rises in Fura-2 loaded HeLa cells. (C) Extracellular alkaline buffer-induced Ca^2+^ increase in Fura-2 loaded HeLa cells was abolished by thapsigargin (1 µM) pretreatment or was inhibited by removal of external Ca^2+^ (Ca^2+^-free HBSS with 4 mM EGTA). (D) ER Ca^2+^ concentration, indicated by the thapsigargin (10 µM)-induced Ca^2+^ increases, was reduced by extracellular alkaline buffers in HeLa cells. (E) Stim1 or Orai1 knockdown inhibited external Ca^2+^ influx induced by extracellular alkaline buffer in NIH3T3 cells. Cells were initially incubated in Ca^2+^-free HBSS with pH adjusted as indicated, followed by 2 mM Ca^2+^ addition. All graphs represent data from three independent experiments. Data quantification in (B), (C), (D), and (E) are presented as mean ± S.E., n = 30–50 cells. The * symbols indicate the results of *t* Test analysis, *p*<0.05.

To further exclude the possibility that SECRA activity might be affected by monovalent cations, such as ammonium or DIEA.H^+^, we used an alternative method to alkalinize pHi by simply raising extracellular pH (Figure 7A) [34]. We found that alkaline extracellular buffer, not acidic buffer, induced cytosolic Ca^2+^ increases in HeLa cells (Figure 7B), which were abolished by thapsigargin pretreatment and diminished in a Ca^2+^ free medium (Figure 7C). We also examined ER Ca^2+^ content at different extracellular pH in HeLa cells (Figure 7D). ER Ca^2+^ contents in alkaline pH buffer (8.0, 8.5 and 9.0) were significantly reduced compared to that in the acidified or neutral pH buffers (6.0, 6.5, 7.0 and 7.4). Thus, extracellular alkalinization also triggers cytosolic Ca^2+^ release from the ER Ca^2+^ pool, as well as induces Ca^2+^ influx. In addition, addition of 2 mM Ca^2+^ in Ca^2+^ free alkaline extracellular buffer induced Ca^2+^ increase through influx, which was markedly inhibited in NIH3T3 cells with Stim1 or Orai1 knockdown (Figure 7E), indicating that alkaline pH buffer-triggered Ca^2+^ influx is via SOCs as well. In summary, our results indicated that extracellular alkaline buffer triggers cytosolic Ca^2+^ increase via intracellular alkalinization as well.

## Discussion

Previous studies on intracellular alkalinization induced Ca^2+^ release from intracellular stores suggested that the ER Ca^2+^ pools are the main target, yet involvement of IP_3_ was a topic of debate [19], [23], [24]. Studies by Danthuluri et al. also showed that intracellular alkalinization increased Ca^2+^ efflux and decreased total cell Ca^2+^ concentration in bovine aortic endothelial cells [19]. Here we showed that intracellular alkalinization directly targeted the ER Ca^2+^ pools in a wide variety of cell types (Figures 2A and S4), but blocking two main calcium releasing channels in the ER, IP_3_Rs and RyRs, failed to affect the alkaline pH-induced Ca^2+^ release (Figures 2B and 2C). Instead, we found that alkaline pHi inhibited the Ca^2+^ refilling activity of ER SERCA, leading to a decrease of the ER Ca^2+^ content (Figure 3). The inhibition of the ER Ca^2+^ refilling by alkaline pHi was also manifested as the retardation of the decay of the histamine or ATP evoked Ca^2+^ transients by intracellular alkalinization (Figure 4). In addition, we showed that the consequence of the alkaline pHi-induced depletion of ER Ca^2+^ pools is the activation of extracellular Ca^2+^ influx via SOCs of Stim1 and Orai1, which contributes to the sustained elevation of the cytosolic Ca^2+^ levels (Figure 5). A previous study also suggested that intracellular alkalinization induces Ca^2+^ influx via SOCs [35]. Taken together, we have provided a clear picture of how alkaline pHi increases cytosolic Ca^2+^ levels: alkaline pHi inhibits SERCA activity to decrease ER Ca^2+^ refilling leading to Ca^2+^ leak from ER and lower ER Ca^2+^ content, then the partially depleted ER Ca^2+^ pools activate SOCs, mediated by Stim1 and Orai1, leading to Ca^2+^ influx. The partial depletion of ER Ca^2+^ pools by intracellular alkalinization via the inhibition of SERCA is similar to that by lower doses of thapsigargin (Figure S12). Yet, it remains to be determined whether alkaline pH affects some poorly studied or uncharacterized Ca^2+^ leak channels located in ER, such as presenilins [36] or sec61 complexes [37].

The molecular mechanisms of intracellular alkalinization induced inhibition of SERCA can be understood from its crystal structures [38], [39], [40]. SERCAs are transmembrane P-type ATPases that transport cytoplasmic Ca^2+^ against its concentration gradient into the lumen of the ER Ca^2+^ stores in exchange for luminal protons, at the expense of ATP hydrolysis. During this process, the conformations of SERCAs switch between the E1 and the E2 states, with preference for binding to cytoplasmic Ca^2+^ and luminal protons, respectively. The phosphorylation (from ATP) on a conserved aspartate residue locks SERCAs in an E1 state bound with Ca^2+^, while the dephosphorylation of the residue, catalyzed by a conserved TGES motif of the actuator-domain switches SERCAs to a E2 state bound with protons, making them ready for exchanging with cytoplasmic Ca^2+^ and starting the next cycle. In cytoplasm, the protonation of a glutamate of the TGES motif is essential for the dephosphorylation of SERCA. It is thus reasonable that intracellular alkalinization could inhibit SERCA by preventing the protonation of the glutamate of the TGES motif at its cytoplasmic region. Inside the ER lumen, four carboxylate residues involved in Ca^2+^ binding in the E1 state are also needed to be protonated in order to release the bound Ca^2+^ and transit to the E2 state [38], [39], [40]. It is also possible that intracellular alkalinization by weak bases might increase the pH of the ER lumen, which can easily partition into the ER, thereby preventing the protonation of some of the four Ca^2+^ binding-carboxylate groups. This would result in inhibiting counter-transport of protons from inside ER lumen for the cytosolic Ca^2+^. Either way could lock SERCA in the phosphorylated E2 state and stop the pump cycle. Indeed, alkaline pH has been shown to reduce the rate of SERCA dephosphorylation in vitro [29]. In addition, Anderden et al showed decades ago that the binding affinity of SERCA for Ca^2+^ at alkaline pH was decreased in vitro [41].

We found that intracellular alkalinization triggers Ca^2+^ entry via SOCs mediated by Orai1 and Stim1 in a wide variety of cells, yet whether alkaline pH directly regulates Orai1 and Stim1 remains to be determined. We have actually found that Ca^2+^ influx triggered by thapsigargin is not affected by alkaline pH (Figure S13), suggesting that alkaline pH does not inhibit SOCs. Interestingly, extracellular acidic buffer has been shown to inhibit SOCs, suggesting that protonation of some residues in Orai1 or Stim1 inhibits the gating of SOCs [42].

Besides SOCs, alkaline pH regulates several plasma membrane Ca^2+^ channels for Ca^2+^ entry [5], [15], [21], [22]. The best known one is the sperm-specific channel, CatSper1, a plasma membrane protein located in the principle piece of the sperm tail. Intracellular alkalinization activates CatSper1 and induces Ca^2+^ influx. The result is an increase in the intraflagellar Ca^2+^, which induces hyperactivated sperm motility and is essential for male fertility [5]. In vascular smooth muscle, intracellular alkalinization activates voltage-dependent Ca^2+^ channels for Ca^2+^ influx and vasoconstriction [3]. Interestingly, alkaline-induced Ca^2+^ entry in A7r5 vascular smooth muscle cells also involves a nonselctive cation channel and is associated with the concomitant inhibition of voltage-gated Ca^2+^ current [43]. We speculate that this non-selective Ca^2+^ channel could be SOCs of Orai1 and Stim1. Along this line, the inhibition of voltage-gated Ca^2+^ current by Orai1 and Stim1 has been elegantly illustrated by two recent studies [44], [45]. Alkaline pH also activates transient receptor potential (TRP) channel V and A for Ca^2+^ entry in neurons, which is related to pain sensation [14], [15]. Moreover, it has been shown that the deprotonation of two cysteine residues in TRPA1 is involved in activation by intracellular alkalization [15]. Besides Ca^2+^ channels, pH affects a number of other ion channels, including K^+^ channels and aquaporins [46]. Without questions, pHi-dependent modifications on ion channels, structure proteins, or signaling modules, play important roles in regulating their functions.

Even a small change of pH could markedly influence cell behavior, which is why both intra- and extra-cellular pH are tightly regulated. Many physiological and pathological conditions produce intra- or extra- cellular alkalinity, which in turn affects a number of cellular processes. Physiologically, intracellular alkalization has been linked to oocyte maturation, sperm activation, cell proliferation, differentiation, migration, and chemotaxis. Pathologically, intracellular alkalization is a hallmark of many tumor cells and is associated with tumor progression [47]. It is also well known that hyperventilation induces respiratory alkalosis [48], and both urinary tract infections and irritable bowel symptoms produce high urinary and blood pH [49]. Although pH can directly affect cellular processes by changing the ionization state of proteins, lipids, or other molecules, the secondary effects of pH changes, such as alkalization-induced cytosolic Ca^2+^ increase as described here, on theses cellular events should also be taken into consideration. For example, during cell cycle progression, alkalization induced by NHE activation is required for G2 to M phase transition by unknown mechanisms [7]. Considering Ca^2+^ signaling is required for G2 to M transition [50], it is conceivable that intracellular alkalization could result in cytosolic Ca^2+^ spikes, as described in this study, facilitating cell entry into M phase.

Oncogene-dependent overactivation of NHE1 is responsible for intracellular alkalization in cancer cells, where alkaline pHi induces cell proliferation independent of serum. The result is producing poorly vascularized yet dense cell masses, which could in turn create a favorable microenvironment for tumor progression and metastasis [2], [51]. It is also well established that Ca^2+^ signaling regulates cell proliferation and differentiation. Dysregulation of Ca^2+^ contributes to tumor development and metastasis [52]. The results in this study establish a clear mechanistic inter-relationship of the pHi and Ca^2+^ and should provide a valuable framework for investigating the over-activated Ca^2+^ signaling activities found in tumors.

## Materials and Methods

### Cell Culture

HeLa, NIH3T3 and 293T cells (*ATCC*) were maintained in DMEM (*Invitrogen*) plus 10% fetal bovine serum (*Invitrogen*) and 100 units/ml of penicillin/streptomycin (*Invitrogen*) at 5% CO_2_ and 37°C. PC12 cells (*ATCC*) were maintained in DMEM plus 7.5% horse serum, 7.5% fetal bovine serum, and 100 units/ml of penicillin/streptomycin at 7.5% CO_2_ and 37°C. The medium was changed every 48 h.

### Intracellular pH measurement

HeLa cells were cultured in 96-well plates at the density of 2×10^4^ cells/well in regular medium overnight. Cells were then incubated with 1 µM BCECF AM (*Invitrogen*), an intracellular pH indicator, in Hank's balanced salt solution (HBSS) at room temperature for 30 min. Afterwards, cells were washed once with HBSS, and pHi of the cells in HBSS at room temperature was measured in TECAN Infinite® 200 plate reader in triplicates with excitations set at 440 nm and 490 nm and emission collected at 530 nm every 3 or 10 second. Emission ratios of two different excitations (490 nm/440 nm) were calculated. In addition, standard intracellular pH curve was obtained by Nigericin/High K^+^ method. Briefly, cells in different wells were incubated in calibration buffer (130 mM KCl, 20 mM NaCl, 5 mM hepes and 10 µM nigericin) with different pHs, 6.6, 7.0, 7.6 and 8.1. The linear pH standard curve was created with defined buffer pH as *X* axis and fluorescence ratio (490 nm/440 nm) as *Y* axis. The intracellular pHs of cells treated with or without drugs were then obtained by calibrating the corresponding 490/440 ratio against the standard curve.

### Intracellular Ca^2+^ measurement

Cells were cultured in 24-well plates at the density of 7×10^4^ cells/well in regular medium overnight and were labeled with 4 µM Fura-2 AM (*Invitrogen*) in HBSS at room temperature for 30 min. The cells were then washed with HBSS three times and incubated at room temperature for another 10 min. Cells were put on the stage of an Olympus inverted epifluorescence microscope and visualized using a 20× objective. Fluorescence images were obtained by alternate excitation at 340 nm and 380 nm with emission set at 510 nm. Images were collected by a CCD camera every 3 or 6 seconds and analyzed by a *Cell R* imaging software.

### Calcium uptake experiment

Ca^2+^ uptake experiments were performed as described previously [53]. Briefly, HeLa cells were trypsinized and washed twice with intracellular buffer (125 mM KCl, 25 mM NaCl, 10 mM Hepes and 0.2 mM MgCl_2_) containing 2 mM EGTA. HeLa cells were then permeabilized with 50 µg/mL saponin in intracellular buffer with 2 mM EGTA. Saponin at this concentration only selectively permeabilized the cell membrane while keeping ER membrane intact. The permeabilization efficiency was checked with trypan blue staining, which was typically over 95%. Next, the cells were washed twice in intracellular buffer to remove saponin and EGTA. Finally, permeabilized HeLa cells were suspended in uptake buffers. Uptake buffers were intracellular buffer plus ATP regeneration system (1 mM ATP, 20 mM creatine phosphate, 20 U/mL creatine kinase) with pH adjusted with HCl/KOH to 6.4, 7.2, or 8.0. Fluo-3 salt (4 µM) was added to uptake buffers to monitor the calcium change. Fluo-3 fluorescence of permeabilized cells in different uptake buffers at 37°C was measured in TECAN Infinite® 200 plate reader with excitation at 488 nm and emission at 526 nm. The fluorescence bleaching was corrected by subtracting the control curve containing no cells.

### Stim1 and Orai1-shRNA lentivirus production and infection

Two optimal 21-mers were selected in the mouse *stim1* and *orai1* gene: CCCTTCCTTTCTTTGCAATAT and CACAACCTCAAC TCGGTCAAA, respectively. Then the two 21-mers were separately subcloned into pLKO.1, a replication- incompetent lentiviral vector for expressing shRNA. Scramble shRNA construct was used as a negative control. 293T cells were used to produce shRNA lentivirus as described previously [25]. For infection, NIH3T3 cells were plated at density of 3×10^5^ cells/well in 6-well plates. Next day, 120 µl of lentiviruses of stim1 shRNA, orai1 shRNA, or scramble shRNA were added to the cells in fresh medium containing 8 µg/ml polybrene. After 24 hrs, cells were selected in fresh medium containing 3 µg/ml puromycin for one week. Knockdown efficiency was verified by quantitative real-time RT-PCR or Western blot analysis.

### Quantitative Real-time RT-PCR

The quantitative real-time RT-PCR using the iScript™ One-Step Kit With SYBR® Green (Invitrogen) was performed normally in Bio-Rad MiniOpticon™ Real-Time PCR Detection System according to the manufacture's instructions. The forward primer for orai1 is 5′ TCCCTGGTCAGCCATAAGAC and the reverse primer is 5′ TCATGGAGAAGGGCATAAGG. Forward primer for GAPDH is 5′ GGACGCATTGGTCG CTGG and reverse primer is 5′ TTTGCACTGGTACGTGTTGAT.

### Western Blot Analysis

Control or shRNA-infected NIH3T3 cells were plated at density of 3×10^5^ cells/well in 6-well plates. Next day, cells were lysed in ice-cold EBC lysis buffer (50 mM Tris-HCl pH8.0, 120 mM NaCl, 0.5% Nonidet P-40, 100 µM NaF, 200 µM Na_3_VO_4_, 100 µg/ml aprotinin, 20 µg/ml leupeptin, 150 µM phenylmethylsulfonyl fluoride, 0.5% sodium deoxy- cholate, and 0.5% SDS). Then the lysates were passed through a 21-gauge needle several times to disperse any large aggregates. Protein concentrations of the cell lysates were determined by the Bradford assay. Proteins (40 µg per lane) were diluted in the standard SDS-sample buffer and subjected to electrophoresis on 10% SDS polyacrylamide gels. Proteins were transferred to an Immobilon-P blotting membrane (Millipore), blocked with 5% milk in Tris-buffered saline (20 mM Tris, 150 mM NaCl, pH 7.6) with 0.1% Tween 20, and incubated with primary antibody against Stim1 (BD Biosciences, 1∶1000 dilution) for 2.5 h. After washing, the blots were probed with a secondary antibody for detection by chemiluminescence.

### Stim1-mCherry and Orai1-EGFP colocalization

Two plasmids, *p*Stim1-mCherry and *p*Orai1-EGFP, were provided by Dr. Gwack, Y [54]. HeLa cells were plated on coverslips in 6-well plates at density of 3×10^5^ cells/well. Next day, *p*Stim1-mCherry and *p*Orai1-EGFP were co-transfected into HeLa cells by Lipofectamine™ 2000. 48 hours after transfection, cells were washed twice with Ca^2+^ free HBSS. Distributions of Stim1 and Orai1 in transfected cells at room temperature were then examined in Ca^2+^ free HBSS containing thapsigargin or DIEA.HBr by confocal laser-scanning microscopy (Olympus FV300) with an Olympus PlanApo 60× Oil objective.

## Supporting Information

Figure S1 **Purification of DIEA.HBr.** (A) HPLC fractionation of an esterification reaction. DIEA.HBr was purified in fraction 7. (B) Chemical structure of DIEA.HBr. (C) DIEA.HBr crystals were obtained by recrystalization method. (D) H-NMR of DIEA.HBR. (E) C-NMR of DIEA.HBr.
(PDF)

Figure S2 **NaBr (4 mM) and KBr (4 mM) cannot induce any cytosolic Ca^2+^ change compared with the effect of DIEA.HBr (4 mM).** The graphs represent data from three independent experiments.
(PDF)

Figure S3 **DIEA.HBr induces cytosolic pH increase in a dose dependent manner in HeLa cells.** The graphs represent data from three independent experiments.
(PDF)

Figure S4 **Intracellular alkalization induced by DIEA.HBr (4 mM) releases Ca^2+^ from ER pools in various cell types, including D3 mouse embryonic stem cells (A), NIH3T3 fibroblasts (B), BHK21 fibroblasts (C), HEK293T cells (D), HL 60 leukemic cells (E), PC 12 cells (F), Jurkat T lymphocyte cells (G), and THP-1 leukemic cells (H).** The graphs represent data from three independent experiments.
(PDF)

Figure S5 **The effectiveness of** ***Xestospongin C*** **, U73122, and ryanodine.** (A) Histamine (10 µM) induced Ca^2+^ rise was markedly inhibited by *Xestospongin C* (10 µM, 30 min pretreatment) and U73122 (10 µM, 15 min pretreatment). (B) Ryanodine (20 µM, 30 min pretreatment) blocked caffeine (10 mM) induced Ca^2+^ rise in PC 12 cells whereas caffeine failed to induce Ca^2+^ increases in HeLa cells.
(PDF)

Figure S6 **Complete depletion of lysosomal Ca^2+^ pools by GPN (50 µM) in HeLa cells.** Fura-2 loaded HeLa cells were treated with GPN (50 µM) to released lysosomal Ca^2+^. Subsequent addition of GPN (50 µM) or bafilomycin A1(0.5 µM) failed to release any more Ca^2+^.
(PDF)

Figure S7 **Inhibition of SERCA ATPase activities by alkaline buffers in vitro.** Top: HEK 293T cell lysates (300 µg) were incubated with anti-SERCA3 antibody (PL/IM430, *Sigma*) pre-bound to protein G beads. The SERCA3 immunocomplexes were then washed by TBS and separated evenly into three different pH Tris buffer (100 mM) at pH 7.5, 8.5, and 9.5, respectively. The ATPase activity of the immunocomplexes in different pH buffers were finally measured by a colorimetric assay for ATPase (*Innova Bioscience*) in a 96-well format and done in triplicates. As a control, boiling the immunocomplexes completely killed the ATPase activity. The graphs represent data from three independent experiments, and data quantification are presented as mean ± S.D., n = 3. Bottom: *Western blot* analysis of SERCA3 in SERCA3 IP complexes in indicated buffers after ATPase assay.
(PDF)

Figure S8 **Intracellular alkalinization induced by DIEA.HBr decreases ionomycin-releasable Ca^2+^ pool in HeLa cells.** After 7 min of DIEA.HBr (4 mM) or MQ pretreatment, ionomycin (5 µM) was used to examine intracellular Ca^2+^ pool content in Ca^2+^ free HBSS containing 2 mM EGTA. The graphs represent data from three independent experiments. Quantifications of ionomycin-induced Ca^2+^ peaks were expressed as mean ± S.E., n = 30–50 cells, *p*<0.05.
(PDF)

Figure S9 **Alkaline pH inhibits thapsigargin-sensitive Ca^2+^ uptake capability in HeLa cells in uptake buffer containing ruthenium red.** Quantifications of Fluo-3 fluorescence at 25 min after drug additions were expressed as mean ± S.D., *p*<0.05. All graphs represent data from three independent experiments.
(PDF)

Figure S10 **Intracellular alkalinization inhibits ER Ca^2+^ store filling after histamine treatment in the presence of a PMCA inhibitor.** (A) Thapsigargin (10 µM) or DIEA.HBr (4 mM) inhibited ER Ca^2+^ refilling after histamine (10 µM) treatment in HeLa cells in the presence of sodium orthovandate (5 mM), a PMCA inhibitor. (B) HeLa cells were first over-loaded with Ca^2+^ by extracellular Ca^2+^ (2 mM) and ionomycin (10 µM) addition. Then the buffer was changed to Ca^2+^ free HBSS, and the removal of over-loaded intracellular Ca^2+^ was significantly inhibited in the presence of sodium orthovanadate (5 mM) compared with that in control.
(PDF)

Figure S11 **Intracellular alkalinization partially depletes ER Ca^2+^ pools in HeLa cells.** Treating the cell with DIEA.HBr (4 mM) around the peak of Ca^2+^ curve induced by thapsigargin (10 µM) failed to cause additional Ca^2+^ rise, whereas adding thapsigargin (10 µM) around the peak induced by DIEA.HBr (4 mM) triggered another Ca^2+^ rise. The graphs represent data from three independent experiments.
(PDF)

Figure S12 **The partial inhibition of SERCA by lower doses of thapsigargin results in partial depletion of ER Ca^2+^ content and subsequent cytosolic Ca^2+^ increase in HeLa cells.** (A) ER Ca^2+^ concentration, indicated by the thapsigargin (10 µM)-induced Ca^2+^ increase, was inhibited by pretreatment of Fura-2 loaded HeLa cells with 10 nM thapsigargin. (B)10 nM thapsigargin also directly induced cytosolic Ca^2+^ increase in HeLa cells.
(PDF)

Figure S13 **Intracellular alkalinization has no affect on SOCE pathway induced by thapsigargin.** In Ca^2+^ free HBSS, thapsigargin (10 µM) was used to completely deplete ER Ca^2+^ pool, then intracellular alkalinization was induced by applying DIEA.HBr (4 mM). The amplitude of Ca^2+^ influx during intracellular alkalinization exhibited no significant differences compared with that in control. The graphs represent data from three independent experiments.
(PDF)
