# Supplementary figures and images for: Intracellular Alkalinization Induces Cytosolic Ca2+ Increases by Inhibiting Sarco/Endoplasmic Reticulum Ca2+-ATPase (SERCA)
Source: PLoS One. 2012 Feb 27;7(2):e31905. doi: 10.1371/journal.pone.0031905 (PMC3288054; doi:10.1371/journal.pone.0031905)

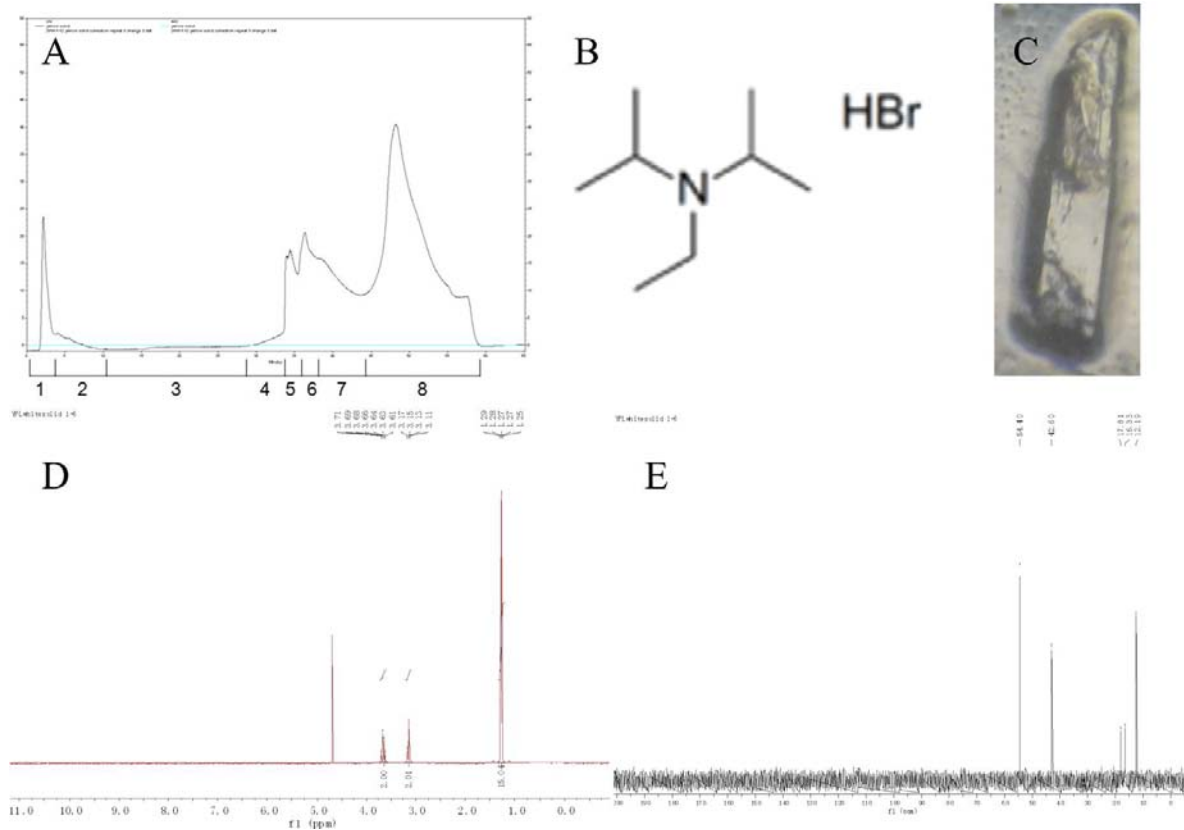

Figure S1

Supplement: Figure S1 — Purification of DIEA.HBr. (A) HPLC fractionation of an esterification reaction. DIEA.HBr was purified in fraction 7. (B) Chemical structure of DIEA.HBr. (C) DIEA.HBr crystals were obtained by recrystalization method. (D) H-NMR of DIEA.HBR. (E) C-NMR of DIEA.HBr. (PDF) [file pone.0031905.s001.pdf]

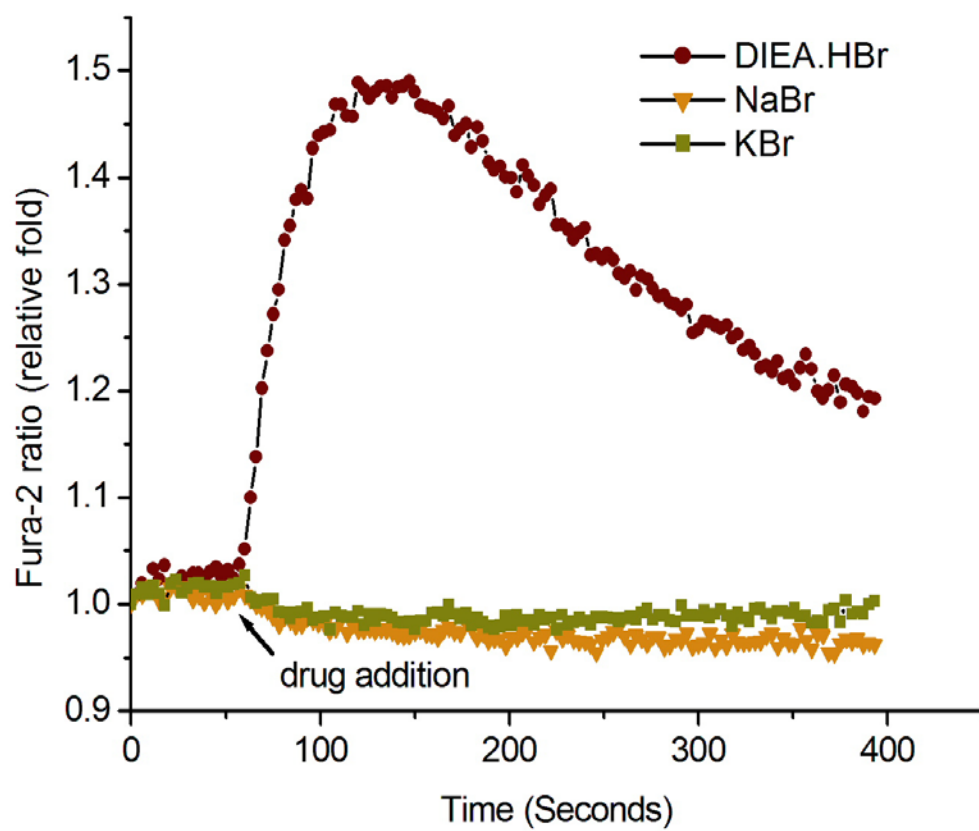

Figure S2

Supplement: Figure S2 — NaBr (4 mM) and KBr (4 mM) cannot induce any cytosolic Ca2+ change compared with the effect of DIEA.HBr (4 mM). The graphs represent data from three independent experiments. (PDF) [file pone.0031905.s002.pdf]

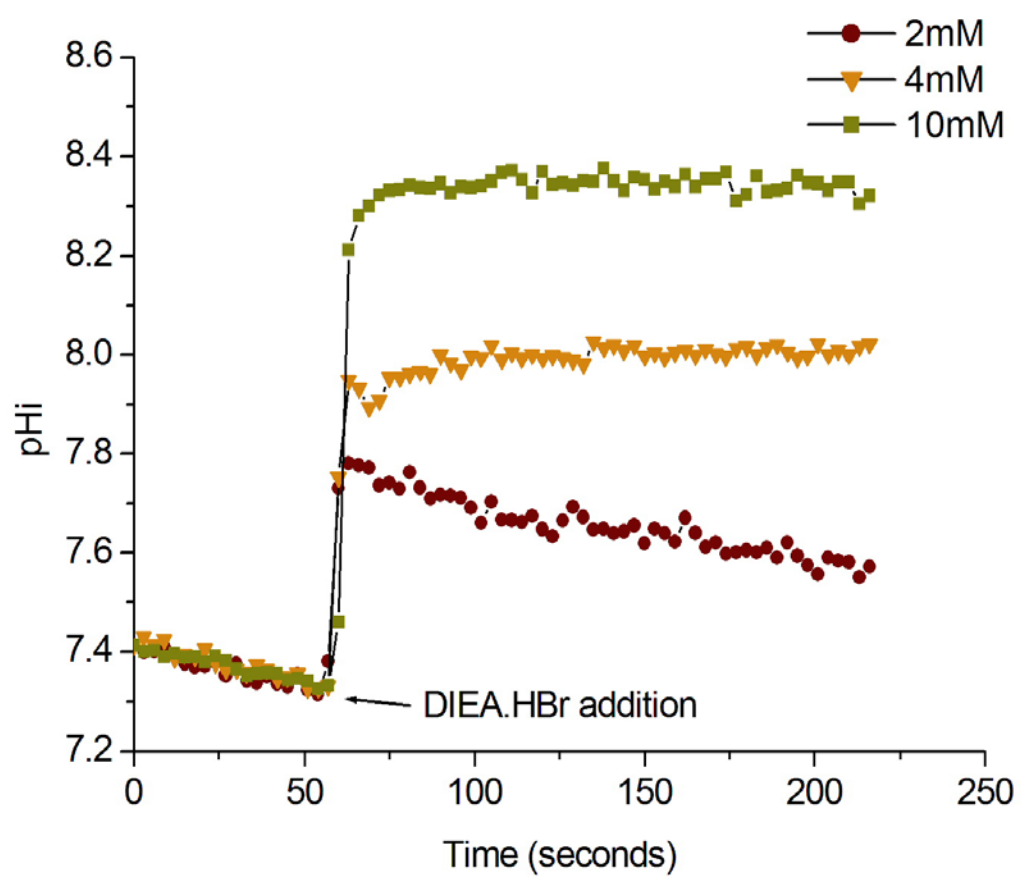

Figure S3

Supplement: Figure S3 — DIEA.HBr induces cytosolic pH increase in a dose dependent manner in HeLa cells. The graphs represent data from three independent experiments. (PDF) [file pone.0031905.s003.pdf]

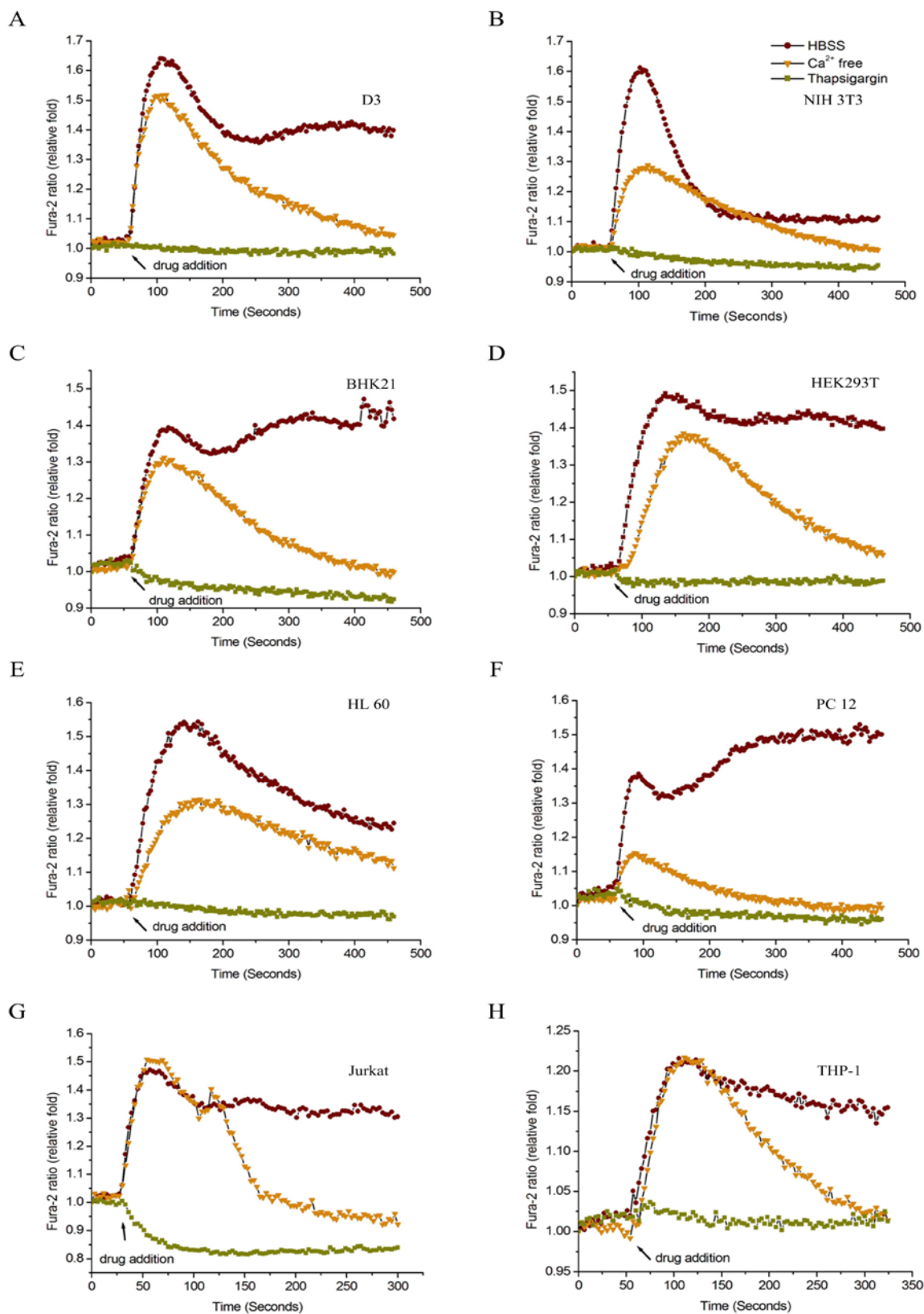

Figure S4

Supplement: Figure S4 — Intracellular alkalization induced by DIEA.HBr (4 mM) releases Ca2+ from ER pools in various cell types, including D3 mouse embryonic stem cells (A), NIH3T3 fibroblasts (B), BHK21 fibroblasts (C), HEK293T cells (D), HL 60 leukemic cells (E), PC 12 cells (F), Jurkat T lymphocyte cells (G), and THP-1 leukemic cells (H). The graphs represent data from three independent experiments. (PDF) [file pone.0031905.s004.pdf]

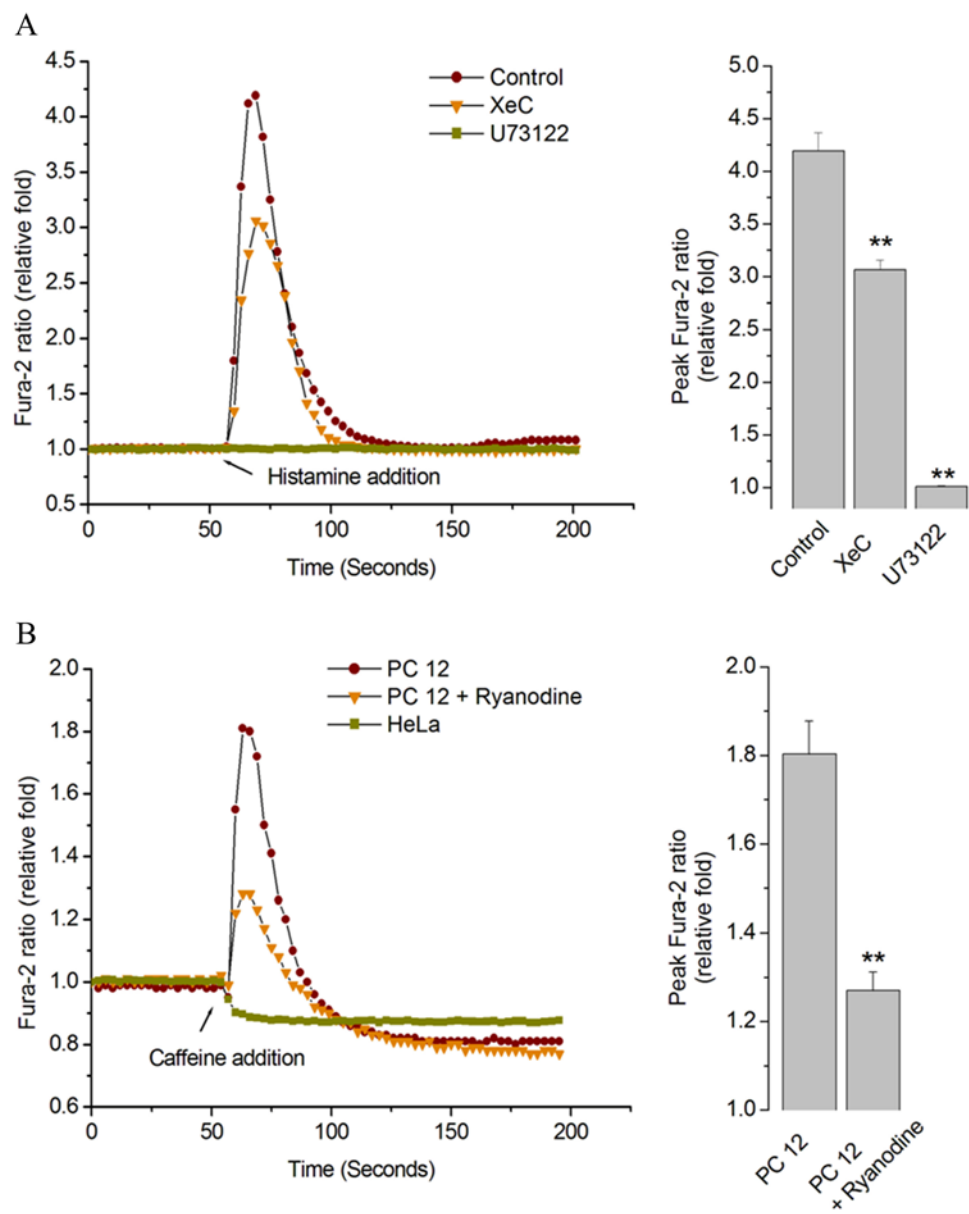

Figure S5

Supplement: Figure S5 — The effectiveness of Xestospongin C , U73122, and ryanodine. (A) Histamine (10 µM) induced Ca2+ rise was markedly inhibited by Xestospongin C (10 µM, 30 min pretreatment) and U73122 (10 µM, 15 min pretreatment). (B) Ryanodine (20 µM, 30 min pretreatment) blocked caffeine (10 mM) induced Ca2+ rise in PC 12 cells whereas caffeine failed to induce Ca2+ increases in HeLa cells. (PDF) [file pone.0031905.s005.pdf]

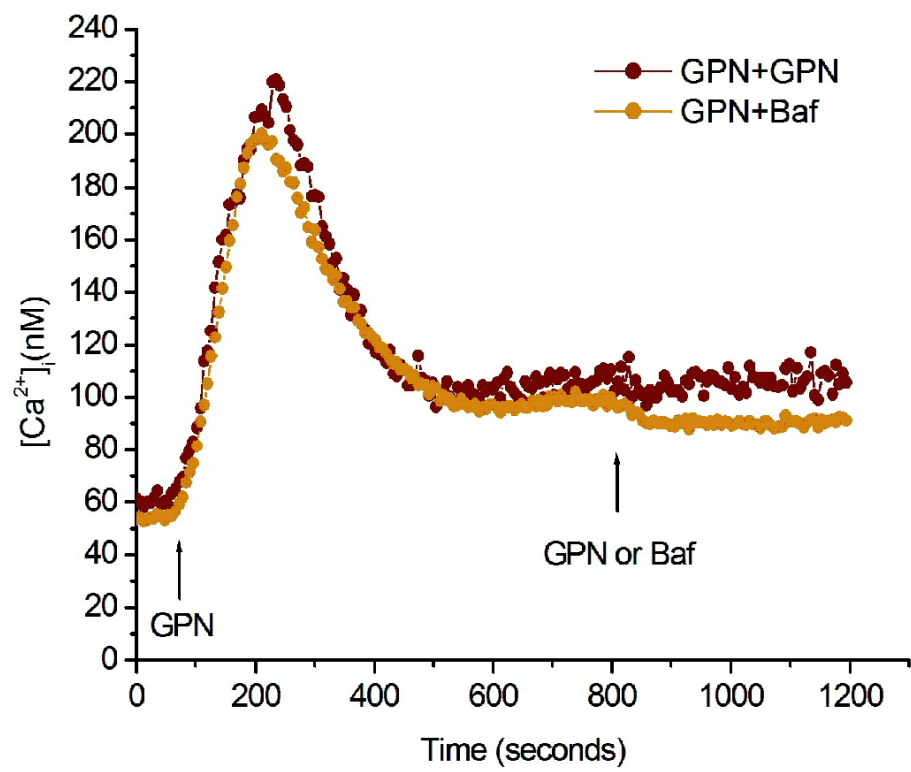

Figure S6

Supplement: Figure S6 — Complete depletion of lysosomal Ca2+ pools by GPN (50 µM) in HeLa cells. Fura-2 loaded HeLa cells were treated with GPN (50 µM) to released lysosomal Ca2+. Subsequent addition of GPN (50 µM) or bafilomycin A1(0.5 µM) failed to release any more Ca2+. (PDF) [file pone.0031905.s006.pdf]

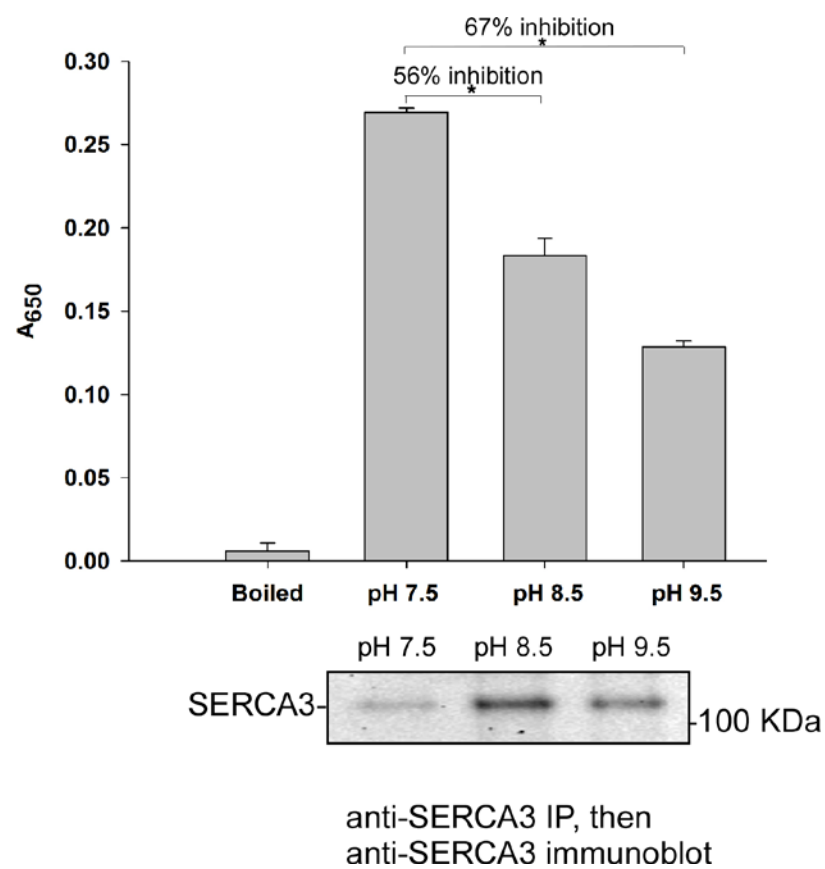

Figure S7

Supplement: Figure S7 — Inhibition of SERCA ATPase activities by alkaline buffers in vitro. Top: HEK 293T cell lysates (300 µg) were incubated with anti-SERCA3 antibody (PL/IM430, Sigma) pre-bound to protein G beads. The SERCA3 immunocomplexes were then washed by TBS and separated evenly into three different pH Tris buffer (100 mM) at pH 7.5, 8.5, and 9.5, respectively. The ATPase activity of the immunocomplexes in different pH buffers were finally measured by a colorimetric assay for ATPase (Innova Bioscience) in a 96-well format and done in triplicates. As a control, boiling the immunocomplexes completely killed the ATPase activity. The graphs represent data from three independent experiments, and data quantification are presented as mean ± S.D., n = 3. Bottom: Western blot analysis of SERCA3 in SERCA3 IP complexes in indicated buffers after ATPase assay. (PDF) [file pone.0031905.s007.pdf]

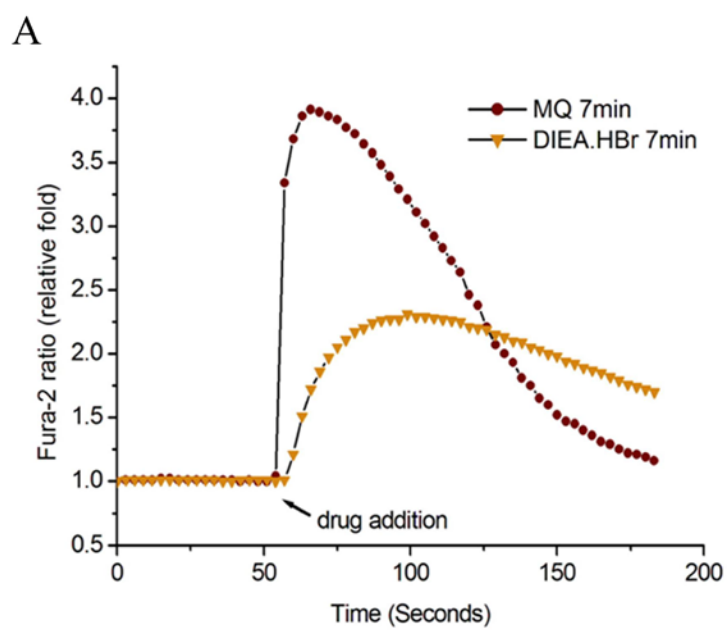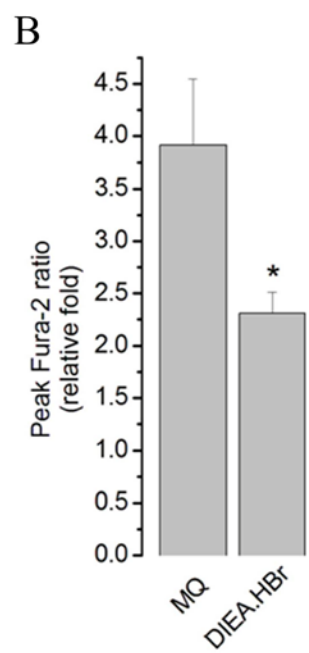

Figure S8

Supplement: Figure S8 — Intracellular alkalinization induced by DIEA.HBr decreases ionomycin-releasable Ca2+ pool in HeLa cells. After 7 min of DIEA.HBr (4 mM) or MQ pretreatment, ionomycin (5 µM) was used to examine intracellular Ca2+ pool content in Ca2+ free HBSS containing 2 mM EGTA. The graphs represent data from three independent experiments. Quantifications of ionomycin-induced Ca2+ peaks were expressed as mean ± S.E., n = 30–50 cells, p<0.05. (PDF) [file pone.0031905.s008.pdf]

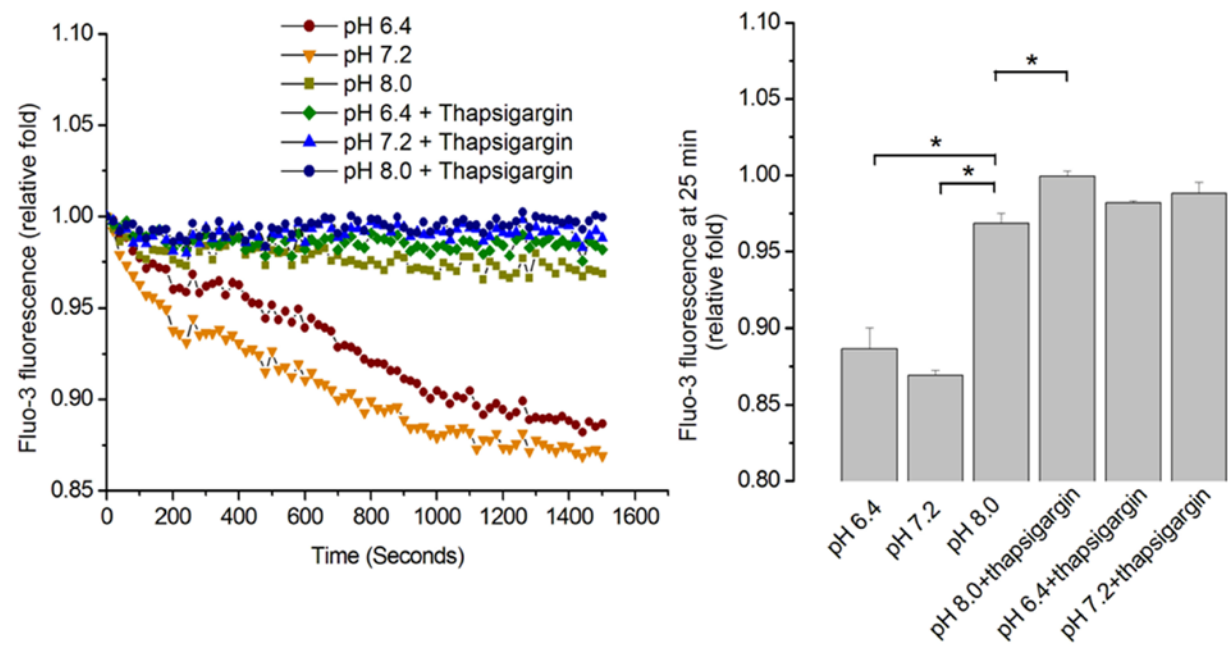

Figure S9

Supplement: Figure S9 — Alkaline pH inhibits thapsigargin-sensitive Ca2+ uptake capability in HeLa cells in uptake buffer containing ruthenium red. Quantifications of Fluo-3 fluorescence at 25 min after drug additions were expressed as mean ± S.D., p<0.05. All graphs represent data from three independent experiments. (PDF) [file pone.0031905.s009.pdf]

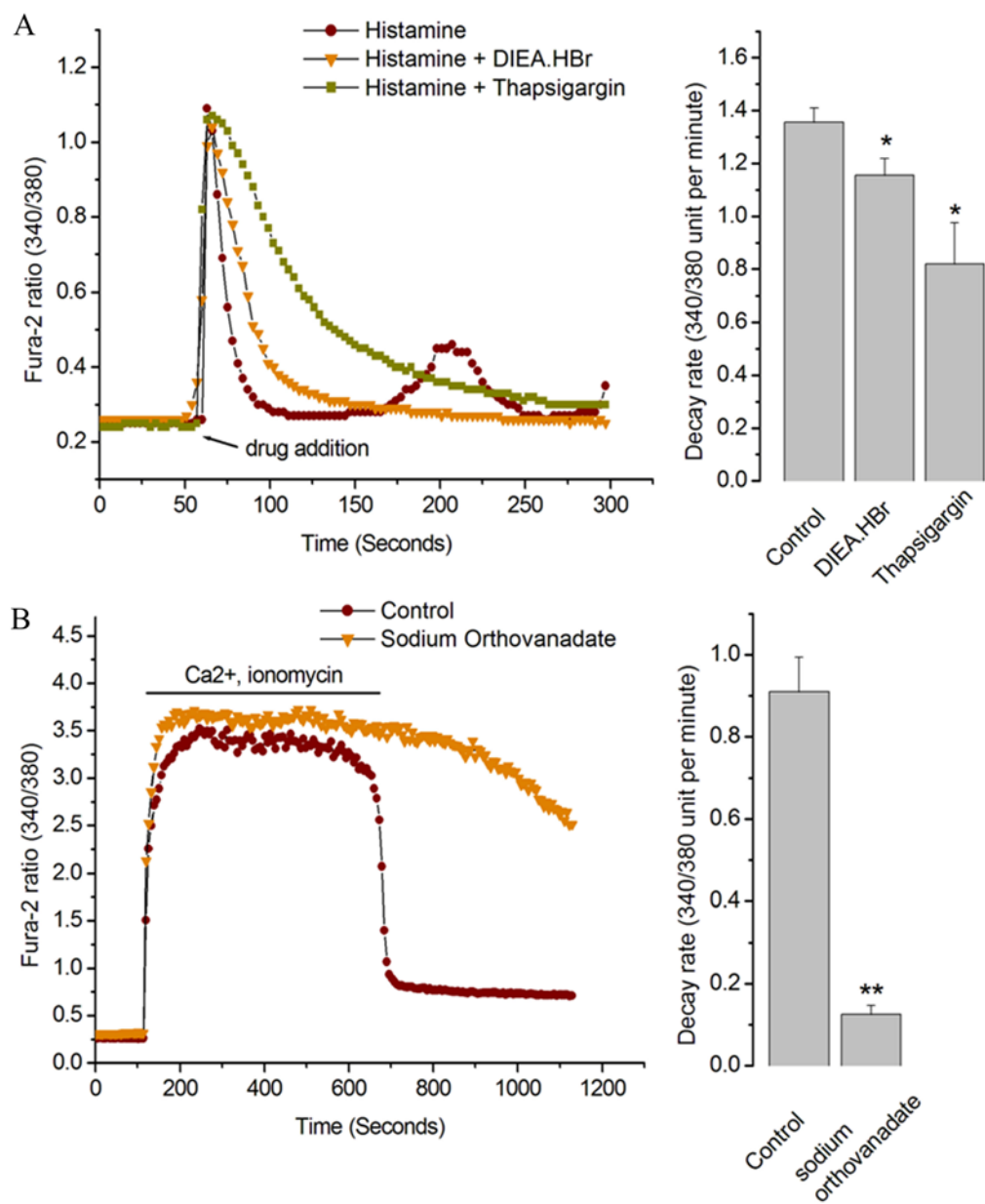

Figure S10

Supplement: Figure S10 — Intracellular alkalinization inhibits ER Ca2+ store filling after histamine treatment in the presence of a PMCA inhibitor. (A) Thapsigargin (10 µM) or DIEA.HBr (4 mM) inhibited ER Ca2+ refilling after histamine (10 µM) treatment in HeLa cells in the presence of sodium orthovandate (5 mM), a PMCA inhibitor. (B) HeLa cells were first over-loaded with Ca2+ by extracellular Ca2+ (2 mM) and ionomycin (10 µM) addition. Then the buffer was changed to Ca2+ free HBSS, and the removal of over-loaded intracellular Ca2+ was significantly inhibited in the presence of sodium orthovanadate (5 mM) compared with that in control. (PDF) [file pone.0031905.s010.pdf]

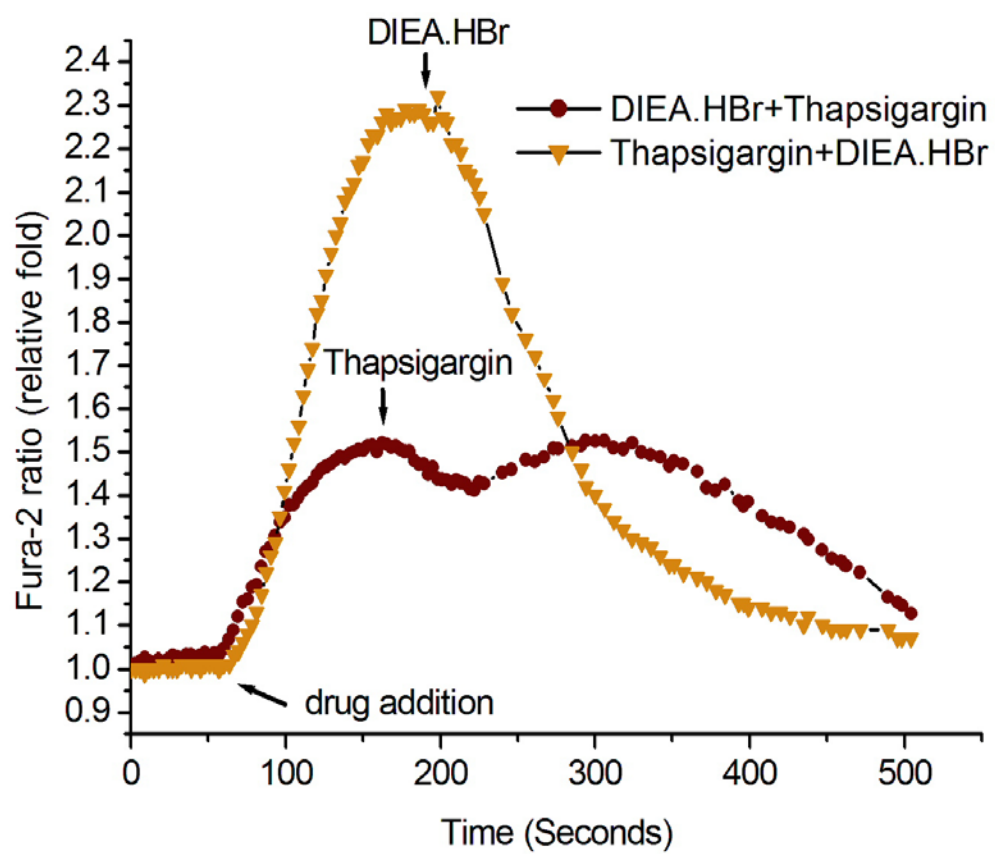

Figure S11

Supplement: Figure S11 — Intracellular alkalinization partially depletes ER Ca2+ pools in HeLa cells. Treating the cell with DIEA.HBr (4 mM) around the peak of Ca2+ curve induced by thapsigargin (10 µM) failed to cause additional Ca2+ rise, whereas adding thapsigargin (10 µM) around the peak induced by DIEA.HBr (4 mM) triggered another Ca2+ rise. The graphs represent data from three independent experiments. (PDF) [file pone.0031905.s011.pdf]

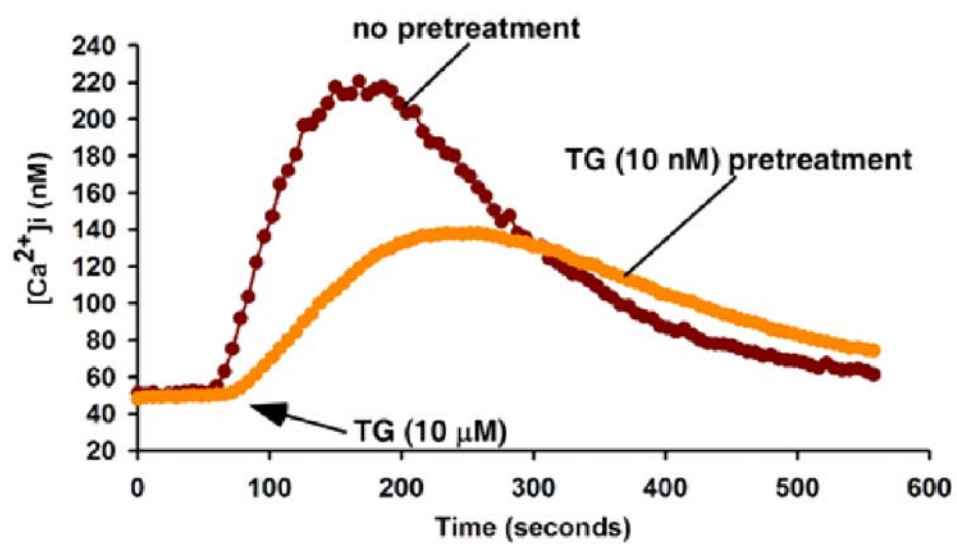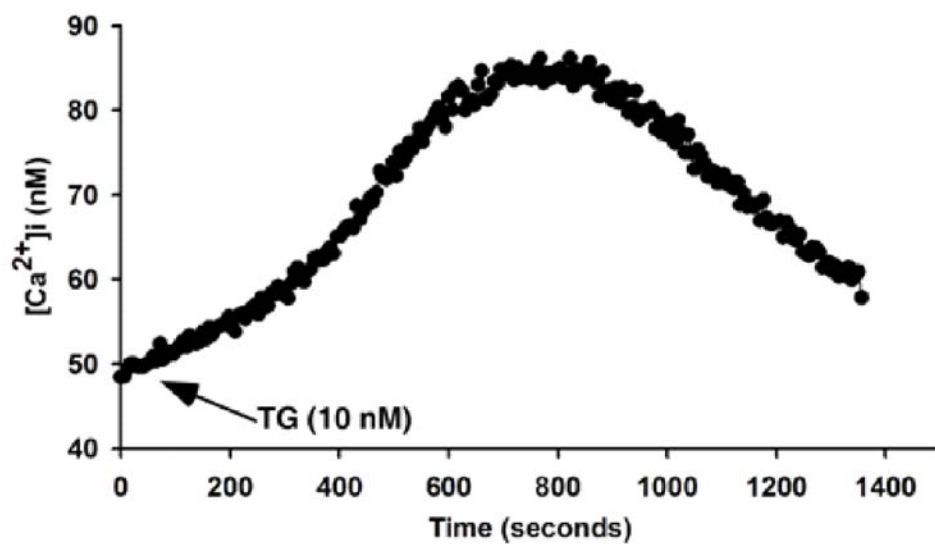

Figure S12

Supplement: Figure S12 — The partial inhibition of SERCA by lower doses of thapsigargin results in partial depletion of ER Ca2+ content and subsequent cytosolic Ca2+ increase in HeLa cells. (A) ER Ca2+ concentration, indicated by the thapsigargin (10 µM)-induced Ca2+ increase, was inhibited by pretreatment of Fura-2 loaded HeLa cells with 10 nM thapsigargin. (B)10 nM thapsigargin also directly induced cytosolic Ca2+ increase in HeLa cells. (PDF) [file pone.0031905.s012.pdf]

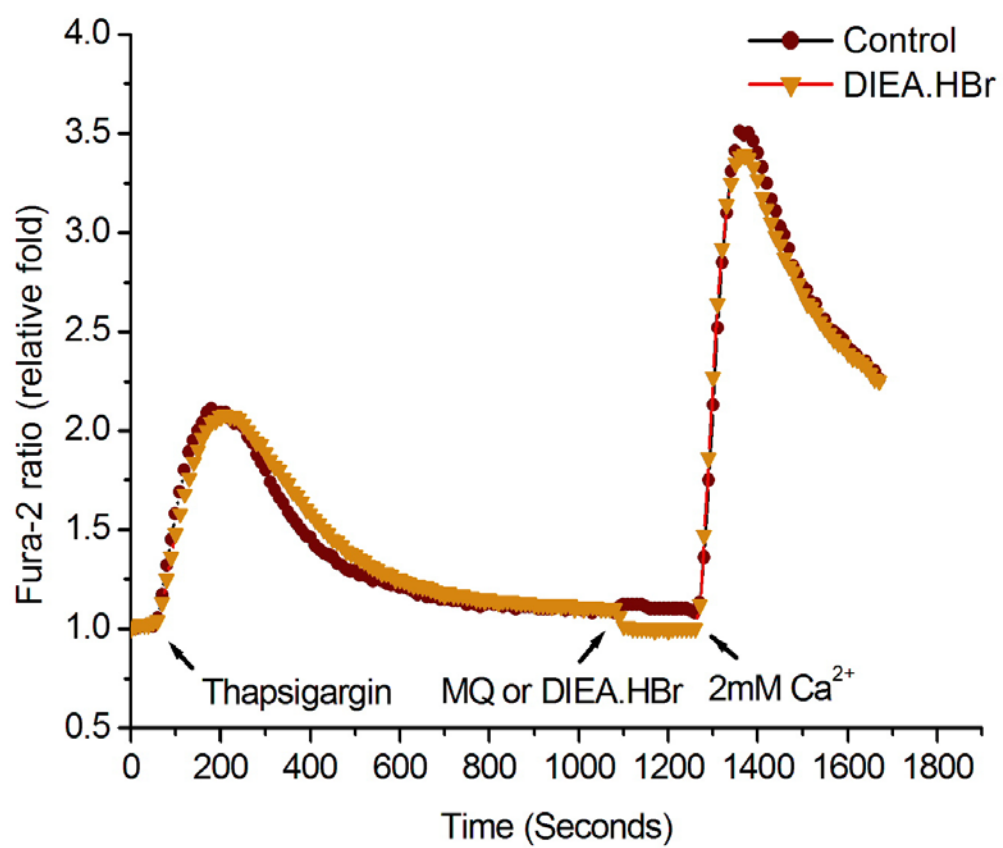

Figure S13

Supplement: Figure S13 — Intracellular alkalinization has no affect on SOCE pathway induced by thapsigargin. In Ca2+ free HBSS, thapsigargin (10 µM) was used to completely deplete ER Ca2+ pool, then intracellular alkalinization was induced by applying DIEA.HBr (4 mM). The amplitude of Ca2+ influx during intracellular alkalinization exhibited no significant differences compared with that in control. The graphs represent data from three independent experiments. (PDF) [file pone.0031905.s013.pdf]
